# Tau‐targeting nanoparticles for treatment of Alzheimer's disease

**DOI:** 10.1002/EXP.20230137

**Published:** 2024-06-21

**Authors:** Shreya Pawar, Mohd Ahmar Rauf, Hosam Abdelhady, Arun K. Iyer

**Affiliations:** ^1^ Use‐inspired Biomaterials and Integrated Nano Delivery (U‐BiND) Systems Laboratory Department of Pharmaceutical Sciences Wayne State University Detroit Michigan USA; ^2^ Department of Surgery Miller School of Medicine University of Miami Miami Florida USA; ^3^ Molecular Imaging Program Karmanos Cancer Institute Detroit Michigan USA; ^4^ Department of Physiology and Pharmacology Sam Houston State University Huntsville Texas USA

**Keywords:** Alzheimer's disease (AD), amyloid, drug delivery, nanoparticles, tau

## Abstract

Alzheimer's disease (AD) is a neurodegenerative disorder characterized by the loss of neural connections and decreased brain tissue volume. Initially affecting the hippocampus and entorhinal complex, which are responsible for memory, the disease later impacts the cerebral cortex, controlling language, logic, and social conduct. While the exact cause is unknown, genetic mutations and environmental factors are implicated. Diagnosis involves computed tomography (CT) scans, Magnetic resonance imaging (MRIs), Positron emission tomography (PET) scans, and lumbar punctures to detect brain abnormalities, protein deposits, and cerebrospinal fluid biomarkers. AD features beta‐amyloid plaques and neurofibrillary tau tangles that disrupt neuronal function, chronic inflammation, blood‐brain barrier impairment, brain atrophy, and neuronal death. There is no cure; current treatments manage symptoms and slow cognitive decline. Research into genetic, cellular, and molecular pathways aims to develop targeted therapies. Tau tangle accumulation is closely linked to AD, making it crucial to explore therapies that restore normal tau pathways and prevent tau accumulation. Nanoparticulate drug delivery technologies offer promise in this area. This review discusses the potential of nanotechnology‐based therapies to target AD‐related tau accumulation and restore normal tau protein mechanics, which could preserve neuronal transmission, synaptic integrity, and brain tissue volume.

## Graphical abstract

Through review of comprehensive literature and research studies, it is found that tau‐targeting nanoparticles may posit beneficial applications to AD therapy. By targeting a key molecular mechanism known to propagate AD pathogenesis, tau‐targeting nanoparticles have the potential to facilitate tau tangle clearance, promote synaptic integrity, and prevent excessive neuronal apoptosis. Further research into tau‐targeting nanoparticles holds significant applications for improving clinical AD outcomes.

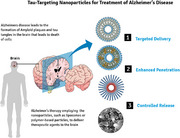

## 1 ALZHEIMER'S DISEASE (AD)

Alzheimer's disease (AD) is an inexorable neurodegenerative condition characterized by the gradual degeneration of neurons, impairment of neuronal connectivity, and ultimately, neuronal demise. Neurons, the fundamental units of the nervous system, play a critical role in facilitating various crucial brain functions such as communication, metabolism, and repair. These intricate neuronal networks form a structural and functional basis for complex brain operations. In the context of AD, it is observed that the damage primarily presents itself in specific brain regions, namely, the hippocampus and the entorhinal complex. These regions are of utmost importance in memory formation. As the pathological progression of the disease occurs, the cerebral cortex, a crucial neural substrate implicated in the intricate processes of language, reasoning, and social behavior, is likewise affected, thereby precipitating a diverse array of cognitive and functional deficits. During the advanced stages of AD, there is pervasive damage across various regions of the brain, accompanied by notable reductions in the volume of brain tissue.

The precise etiology of AD remains predominantly enigmatic; however, scholarly investigations have yielded certain insights regarding putative determinants that may contribute to its pathogenesis. Genetic mutations play a substantial role in the manifestation of early onset AD. Conversely, late‐onset AD is characterized by a multifaceted interplay between genetic predisposition, environmental factors, and lifestyle choices. Various factors have been implicated in the progression of AD.[Supplementary-material exp2341-supitem-0001]

The APOE gene, specifically the APOE ε4 allele, has been identified as a well‐established genetic susceptibility factor for late‐onset AD, contributing to an increased risk of disease onset.^[^ ^4^] Nevertheless, it is imperative to acknowledge that the presence of this particular gene does not ensure the manifestation of AD, as individuals lacking this gene can still be vulnerable to ailments.

Notably, individuals diagnosed with Down syndrome, characterized by the presence of an additional copy of chromosome 21, exhibit a significantly heightened susceptibility to the development of AD.^[^ ^1^, ^2^ ^]^ The genetic locus located on chromosome 21 harbors a gene responsible for the synthesis of amyloid proteins that have been identified as a significant causative agent of AD pathogenesis.

Significant advancements have been made in the comprehension of AD^[^ ^1^, ^2^, ^3^, ^4^, ^5^ ^]^; however, the intricate and multifaceted characteristics of this ailment continue to present persistent obstacles for both researchers and healthcare practitioners. Ongoing endeavors in scholarly investigation and timely identification hold paramount significance in the advancement of efficacious therapeutic modalities and interventions aimed at enhancing the quality of life of individuals afflicted with AD.

## 2 DIAGNOSIS OF AD

**FIGURE 1:**
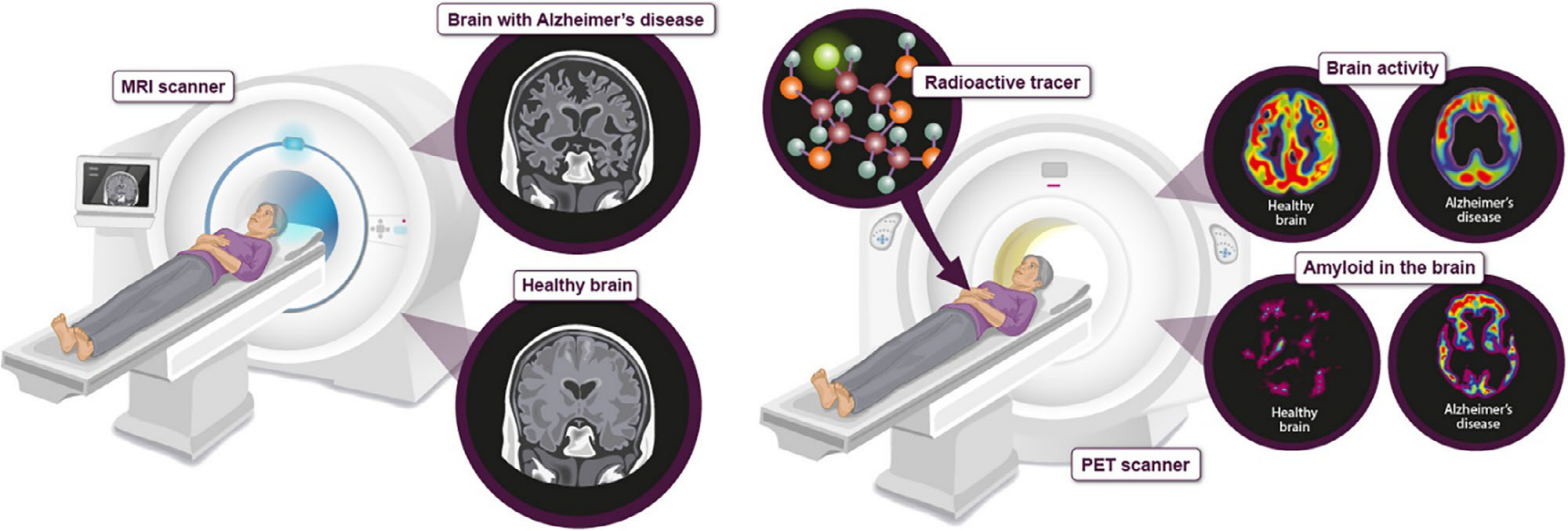
Schematic diagram showing the MRI and PET scanning diagnostic practices and their visualization patterns of healthy brains and Alzheimer's Disease brains. Adapted with permission.^[^ ^15^ ^]^ Copyright 2022, Alzheimer's reserach U [Accessed: June 10, 2022].

In the context of Alzheimer's disease (AD), cognitive impairment extends beyond memory deficits and encompasses difficulties in lexical retrieval, visual processing, spatial orientation, reasoning, and judgment. Memory impairment is a prominent feature of AD. In dementia diagnosis, including AD, various imaging techniques are employed. CT scans are useful for examining changes in cerebral dimensions, comparing them to previous scans or expected sizes for individuals of similar age and physique. Magnetic Resonance Imaging (MRI) plays a crucial role in identifying brain atrophy and other cerebral anomalies. Fluorodeoxyglucose positron emission tomography (FDG‐PET) scans quantify glucose uptake in the brain, revealing reduced levels in specific regions of dementia patients, making them valuable for diagnosis. Amyloid positron emission tomography (PET) scans are used to detect and quantify abnormal accumulations of beta‐amyloid protein, a hallmark of AD. Tracers like florbetapir, flutemetamol, florbetaben, and Pittsburgh compound B are commonly administered for amyloid PET scans. Tau PET scans, using tracers like AV‐1451, PI‐2620, and MK‐6240, effectively identify pathological tau protein aggregates, aiding in AD progression monitoring [Figure 1/Table 1].

**TABLE 1:** Summary of conventionally used AD diagnosis techniques.

Diagnosis technique	Mechanism	AD characteristics identified
Computerized tomography (CT) scan	CT scans utilize an integrative approach with motorized X‐ray sources and advanced mathematical methods to construct 2D cross‐sectional images of the patient. Using a computer, the 2D images can then be coupled to create advanced 3D images of biological systems.	CT scans can identify regions of brain shrinkage and amyloid plaques. Additionally, CT scans are utilized to confirm that other brain abnormalities such as, tumors, subdural hematomas, and strokes are not the source of AD symptoms
Magnetic resonance imaging (MRI)	MRIs implement a highly potent magnetic field to stimulate protons in the body to orient towards the applied magnetic field. Once the radiofrequency field initially applied is removed, MRI detectors measure the energy released by the protons to reorient with the magnetic field. Biological tissue varies across systems, with each having their own unique magnetic properties.	MRIs can show regions afflicted with brain atrophy and detailed images of blood vessels in the brain. Furthermore, MRIs are employed to confirm that bleeding in the brain and fluid build‐up are not contributing to the appearance of AD symptoms.
Positron emission tomography (PET) scan	PET scans carry out their function by utilizing an injected radioactive isotope that emits positrons. The emitted positrons then collide with electrons present in biological tissue to form two photons. These photons are detected by scintillation crystals integrated within the PET scanner. The scintillation crystals absorb the photons and then convert the light energy derived from the photons into applicable electrical signals.	Amyloid PET scans measure the pathological accumulation of beta‐amyloid proteins. Tau PET scans identify the abnormal accumulation of tau proteins. Fluorodeoxyglucose (FDG) PET scans quantify energy utilization in the brain. Widespread studies have indicated that dementia patients often show decreased glucose consumption in certain regions of the brain.
Lumbar punctures	Lumbar punctures are performed by inserting a hollow needle into the subarachnoid space of the spinal column to collect samples of CSF.	Lumbar punctures can identify abnormal levels of beta‐amyloid 42, tau, and phospho‐tau in CSF. All of these proteins are critical AD biomarkers.

Lumbar punctures are commonly utilized in the identification of cerebrospinal fluid (CSF) biomarkers for AD, including beta‐amyloid 42, tau, and phospho‐tau.^[^ ^6^, ^7^ ^]^ Blood tests represent an additional modality for the diagnosis of AD, with the primary objective of detecting brain‐derived biomarker proteins such as beta‐amyloid 42/beta‐amyloid 40 and phospho‐tau 181. The present diagnostic methods for AD exhibit a degree of predictability; however, their sensitivity and accuracy may be enhanced to enable more precise and early detection of AD. This can be achieved through the integration of advanced technologies, such as nanoparticles, with existing imaging agents and tracers. The aforementioned advancements possess the potential to expedite the initiation of therapeutic interventions, thereby potentially impeding the progression of the disease and mitigating the symptoms associated with AD.

Recent studies have been focused on developing integrative methods with nanoparticles to enhance current diagnostic procedures. One study by Razzino et al., synthesized gold‐polyamidoamine dendrimer nanocomposites conjugated with screen‐printed carbon electrodes (SPCE) to facilitate enhanced diagnosis of AD.^[^ ^8^ ^]^ The nanocomposites were also functionalized with anti‐tau antibodies, allowing tau tangles to aid as the target for diagnosis of AD. In the study by Kim et al., long gold‐nanoparticle based nanorods were found to detect tau levels at a significantly low dose of 23.6 Fm.^[^ ^9^ ^]^ One limitation of tau‐targeting gold‐nanoparticle biosensors is the difficulty of the tau epitopes to be accessible to the biosensor. The hydrophobic bonds between tau proteins and the hydrogen bonding between tau epitopes often limit the ability of nanoparticle‐based biosensors to detect the tau protein. This challenge can be overcome by coupling the nanoparticle‐based biosensor with chaotropic agents to hinder tau‐tau interactions. With this, the nanoparticles‐based biosensors were able to detect significantly low tau concentrations of 0.1 pm, compared to the 1.0 pm tau concentration detected by the nanoparticle‐based biosensor without the chaotropic agents. Although these studies have found success in implementing gold nanoparticles as in vitro biosensing platforms, further research is necessary to establish their biocompatibility, administration, and bioaccumulation in vivo*. *Another recent study fabricated magnetic nanoparticles coated with dextran to detect significant AD blood biomarkers, such as amyloid‐beta and phosphorylated tau.^[^ ^10^ ^]^ This was accomplished by functionalizing the surface of the magnetic nanoparticles with antibodies against amyloid‐beta and phosphorylated tau. Further studies showed that the synthesized magnetic nanoparticles had high specificity and sensitivity when differentiating between the healthy control group and groups with mild cognitive impairment and AD dementia. However, the magnetic nanoparticles had moderate specificity and sensitivity when differentiating between the mild cognitive impairment and AD dementia groups. In the study by Chen et al., success was found in implementing CuInS_2_/ZnS quantum dots functionalized with dopamine for the detection of tau protein.^[^ ^11^ ^]^ Results showed that the synthesized quantum dots had the ability to detect tau protein concentrations as low as 9.3 pm. Another study synthesized graphene oxide magnetic nanoparticles conjugated with tannin‐capped silver nanoparticles to detect tau tangles.^[^ ^12^ ^]^ These nanoparticles were able to detect low tau concentrations of 5.74 fg mL^−1^. Multi‐walled carbon nanotubes were also found to detect significantly low tau concentrations of 15.0 nm in artificial cerebrospinal fluid and 7.8 nm in solution buffer.^[^ ^13^ ^]^ Another study showed that surface plasmon resonance systems synthesized with silica fibers had promising potential to detect total‐tau protein levels as low as 2.4 pg mL^−1^ and phosphorylated‐tau protein levels as low as 1.6 pg mL^−1^.^[^ ^14^ ^]^ Specifically, these nanoparticulate systems effectively differentiated between healthy controls and AD patients and showed a 6‐fold increase in total‐tau levels and a threefold increase in phosphorylated‐tau levels between AD patients and the healthy controls.

As shown by extensive studies, tau‐targeting nanoparticles show promising potential to function as an effective AD diagnostic tool. However, before these nanoparticulate systems can be utilized on a widespread clinical scale, certain limitations such as efficiency, biosensor multiplexing, long‐term biocompatibility and cost‐effective manufacturing must be overcome. Overall, coupling nanoparticle‐based diagnostics with conventional diagnostics could significantly enhance the efficacy of AD biosensors and offer improved AD diagnostic techniques.

## 3 CELLULAR CHANGES ASSOCIATED WITH AD

The course of AD and its pathophysiology are both significantly affected by cellular alterations that are linked to the disease. Plaques can develop in the brain as a result of the accumulation of β‐amyloid proteins, which are produced from amyloid precursor proteins. Plaques can affect neuronal communication as well as the functioning of cells, which can contribute to cognitive impairment in AD.

**FIGURE 2:**
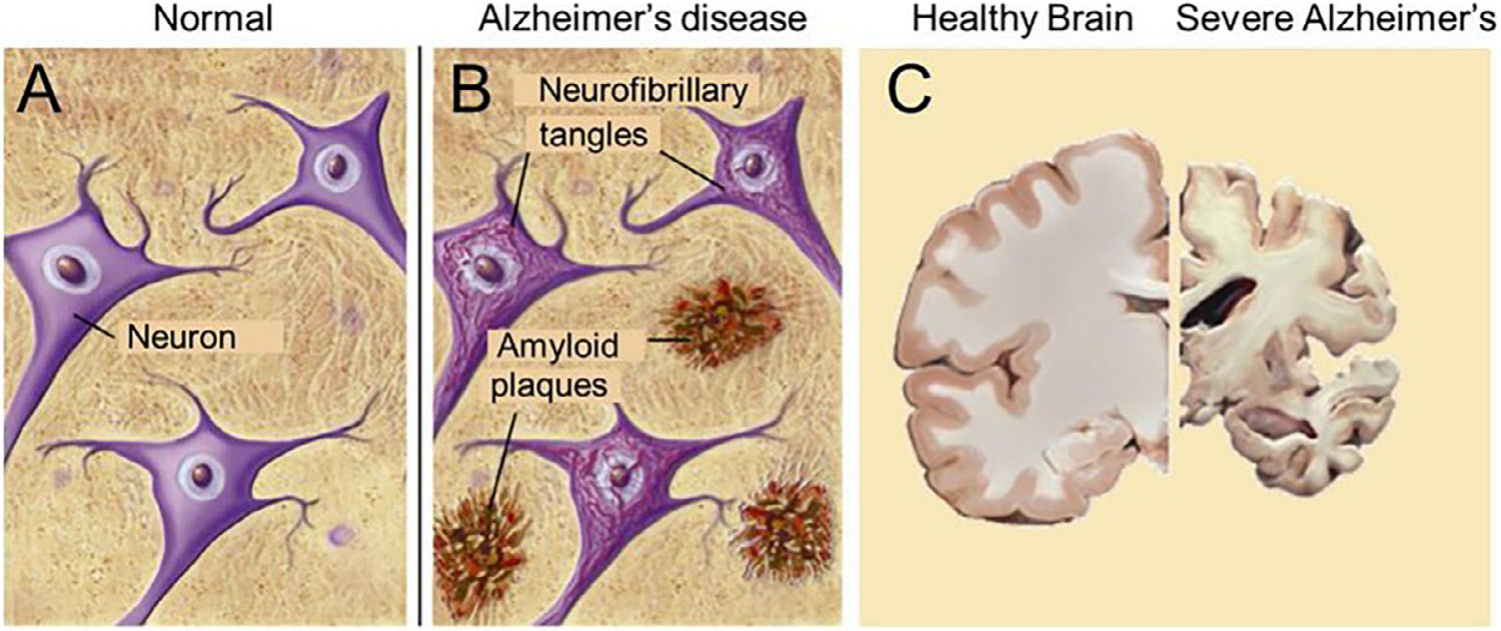
(A) Neurons, neuronal networks, and other corresponding neuroanatomical structures are all functional and have the appropriate structural integrity in normal brains. (B) In AD brains, amyloid plaques form between neurons and disrupt neuronal networks and communication. In addition, neurofibrillary tangles form within neurons to interfere with their structure and function. (C) AD brains undergo pathological changes that significantly reduce brain tissue volume. Reproduced under the terms of the CC‐BY 4.0 license.^[^ ^20^ ^]^ Copyright 2019, The Author(s), published by LIDSEN Publishing Inc.

The growth of neurofibrillary tau tangles is another characteristic of AD. Tau is a protein that helps to maintain the structural integrity of microtubules in healthy neurons.^[^ This, in turn, facilitates the transport of chemicals and nutrients by neuronal microtubules. On the other hand, tau detaches from microtubules and aggregates to create tangles inside neurons when AD is present. These tau tangles disrupt the transfer of nutrients and chemicals, which ultimately results in a breakdown of synaptic communication and memory impairment. Significant research has shown that the widespread loss of synaptic communication can negatively impact normal cognitive processes. Thus, targeting tau tangles and facilitating their degradation possess great therapeutic potential. With a successful tau‐targeting intervention system, nutrient transfer, synaptic communication, and memory can possibly be preserved in AD patients (See Figure 2).

AD is characterized by the widespread presence of chronic inflammation in the brain.^[^ ^16^, ^17^, ^18^ ^]^ The removal of waste and toxins from the brain is the responsibility of microglia, which are the immune cells found in the brain. In contrast, AD causes microglia to become less effective at removing beta‐amyloid plaques and tau tangles, which may be caused by a defect in the TREM2 gene. In AD, astrocytes, which also play a role in sweeping away waste in the brain, are unable to function properly. The buildup of microglia and astrocytes in neurons leads to the production of chemicals, which contribute to the course of AD by causing persistent inflammation and neuronal damage. Thus, interventional systems designed to facilitate tau tangle clearance may help alleviate the excessive inflammation and inflammatory‐mediated neuronal damage in AD patients.

Patients with AD often have problems with the blood‐brain barrier (BBB), which makes it more difficult for the brain to sweep out tau tangles and amyloid plaques and prevent glucose from entering the brain.^[^ ^19^ ^]^ It is possible that these changes, together with brain shrinkage, the loss of synaptic connections, and the death of neurons, occur years before memory and cognitive difficulties become apparent. This AD pathological mechanism further supports that tau‐targeting interventional systems possess significant potential to restore normal neuronal mechanics and facilitate synaptic connection integrity, adequate brain volume, neuronal viability, and normal cognition.

It is essential to understand these cellular alterations to create targeted therapeutics that target the underlying mechanisms of AD and could potentially slow down or stop the progression of the disease. In the future, it is hoped that research focusing on comprehending these complicated processes may lead to more effective treatments and even prevention of AD. For example, understanding the critical pathways leading to tau phosphorylation and aggregation can foster the development of drug compounds focused on downregulating proteotoxic tau functions. Furthermore, integrating these novel therapeutics with nanotechnology may further amplify their beneficial effects by improving their bioaccumulation, enhancing biocompatibility, increasing circulation time, enabling targeting to diseased tissue, and facilitating controlled drug release.

## 4 CURRENT TREATMENTS OF AD

The treatments that are now available for AD are restricted to symptom management and delaying cognitive loss; nevertheless, they do not offer a cure for the condition.^[^ ^21^ ^]^ Patients diagnosed with Alzheimer's disease are often given medications to help retain mental function and reduce memory loss.^[^ ^22^ ^]^ Some examples of these medications include donepezil, rivastigmine, and memantine. Donepezil blocks the action of an enzyme called cholinesterase, which stops the breakdown of acetylcholine in the brain.^[^ ^21^, ^22^ ^]^ Rivastigmine prevents the breakdown of acetylcholine as well as butyrylcholine, whereas memantine obstructs the deleterious effects of glutamate and regulates its activation. Both of these effects are caused by the accumulation of glutamate in the body.

It is difficult to identify clinical trial subjects for possible therapeutic drugs which is one of the reasons why AD is so difficult to cure. Another reason for the difficulty of treating AD is that the biochemical processes underlying AD pathogenesis are not completely understood. At this point in time, the primary objective of AD research is the development of treatments that inhibit the progression of the disease by focusing on genetic, cellular, and molecular pathways. For instance, therapies now in development focus on targeting beta‐amyloid proteins, tau clumps, and phosphorylation of tau. Overall, these medicinal therapies aim to preserve cerebrovascular function, synaptic transmission, and availability of essential neurotransmitters. Utilizing nanoparticulate systems as a means of delivering drugs in a targeted manner enables these exploratory treatments to exert an even greater beneficial impact on patients.

## 5 CLINICAL TRIALS

Clinical trials are of paramount importance for the progression of knowledge and therapeutic interventions for AD. In recent decades, several clinical trials have been undertaken to identify efficacious therapeutic interventions and diagnostic tools for AD.^[^ ^23^ ^]^ Tau protein has been identified as a pivotal focal point in numerous clinical trials.

Numerous diagnostic clinical trials have been conducted to investigate the efficacy of brain imaging techniques that specifically target tau proteins to enhance early detection and monitoring of AD.^[^ ^24^ ^]^ The primary objective of these trials was to gain a comprehensive understanding of the pathogenic pathways underlying AD. This entails investigating the intricate relationship between tau, a protein associated with AD pathology, and other biomarkers. Furthermore, these trials sought to evaluate the specific role of tau in the progression of AD. By elucidating these fundamental aspects, we aimed to enhance our knowledge of AD pathogenesis and identify novel therapeutic targets.

Within the realm of therapeutic trials, significant emphasis has been placed on interventions that specifically target tau regulatory processes, with particular attention directed towards the phenomenon of hyperphosphorylation. The present trial aimed to assess alterations in tau protein concentrations to ascertain the effectiveness of the interventions under investigation in mitigating tau pathology and enhancing cognitive function.

The findings derived from the aforementioned clinical trials are currently undergoing meticulous scrutiny to evaluate the viability of incorporating interventions into a comprehensive strategy for the treatment of AD on a large scale. It is imperative to acknowledge that a considerable number of these trials are currently situated within their preliminary stages, necessitating a comprehensive investigation prior to the authorization of any intervention for clinical application. Ongoing endeavors in the field of clinical research play a pivotal role in the treatment and management of AD. Although a significant knowledge gap remains, ongoing clinical trials offer a promising outlook for the identification of efficacious therapeutic interventions and diagnostic tools to address the formidable complexities associated with this neurodegenerative disorder.

## 6 INTRODUCTION TO TAU PROTEINS

**FIGURE 3:**
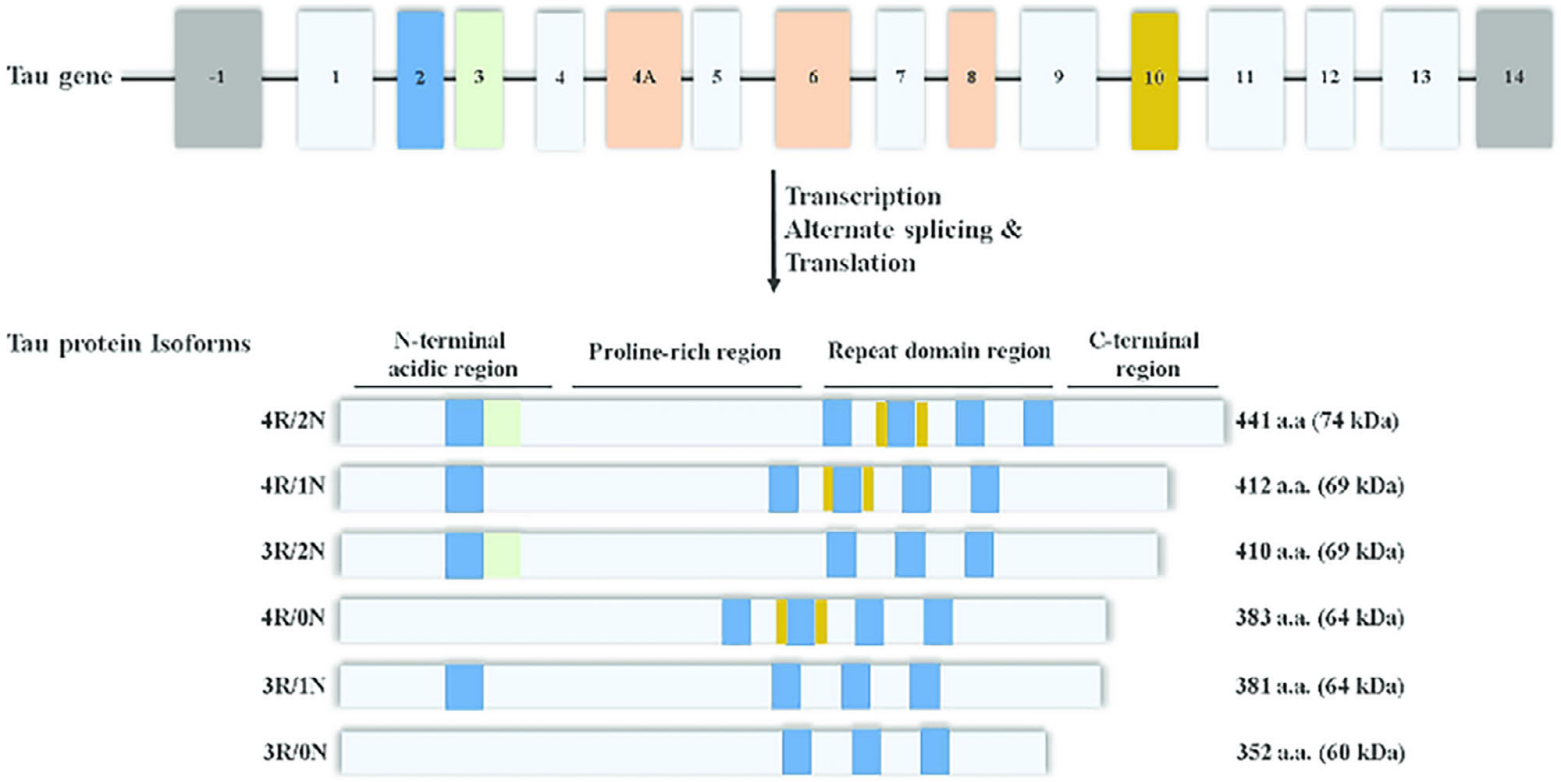
Transcription, post‐transcriptional modifications, and translation of tau protein from tau MAPT gene. Once the tau protein is transcribed, it undergoes alternative splicing and translation to form six different possible isoforms. Three isoforms have three binding domains, while the other three isoforms have four binding domains. The regions (N‐terminal, proline‐rich, repeat domain, and C‐terminal) are all shown on each tau protein isoform. Adapted under the terms of the Creative Commons by Attribution (CC‐BY) license.^[^ ^31^ ^]^ Copyright 2016, The Author(s), published by MDPI.

Tau proteins are essential components of nerve cells and play an important role in preserving the structural integrity of microtubules. Microtubules are required for the intracellular transport and organization of cellular structures. Alternative splicing of MAPT, which is located on chromosome 17q21.3, is the primary mechanism by which these proteins are created.^[^ ^25^, ^26^ ^]^ Tau proteins interact with tubulin to stabilize microtubules and govern their assembly, dynamic activity, and spatial organization.^[^ ^26^ ^]^ These processes are controlled by the spatial structure of the microtubules. They are especially active in the distal regions of axons, where they increase microtubule flexibility by controlling phosphorylation.^[^ ^25^ ^]^ This helps the axons transmit signals more efficiently (Figure 3).

The development of tangled filaments is a characteristic feature of AD, which is caused by the hyperphosphorylation of tau proteins. These tau tangles are a hallmark of the etiology of AD and contribute to the increasing neurodegeneration that is characteristic of the condition. All six isoforms of tau protein are hyperphosphorylated in the brain tissue of patients diagnosed with AD.

Tau proteins can be found in a number of different isoforms, with three isoforms comprising three binding domains and the other three comprising four binding domains.^[^ ^27^ ^]^ These binding domains make it easier for tau to engage directly with microtubules, which in turn stimulates microtubule polymerization and helps maintain the dynamics under control. In addition, tau proteins have a positive charge, which causes them to be drawn to microtubules that have a negative charge.

**FIGURE 4:**
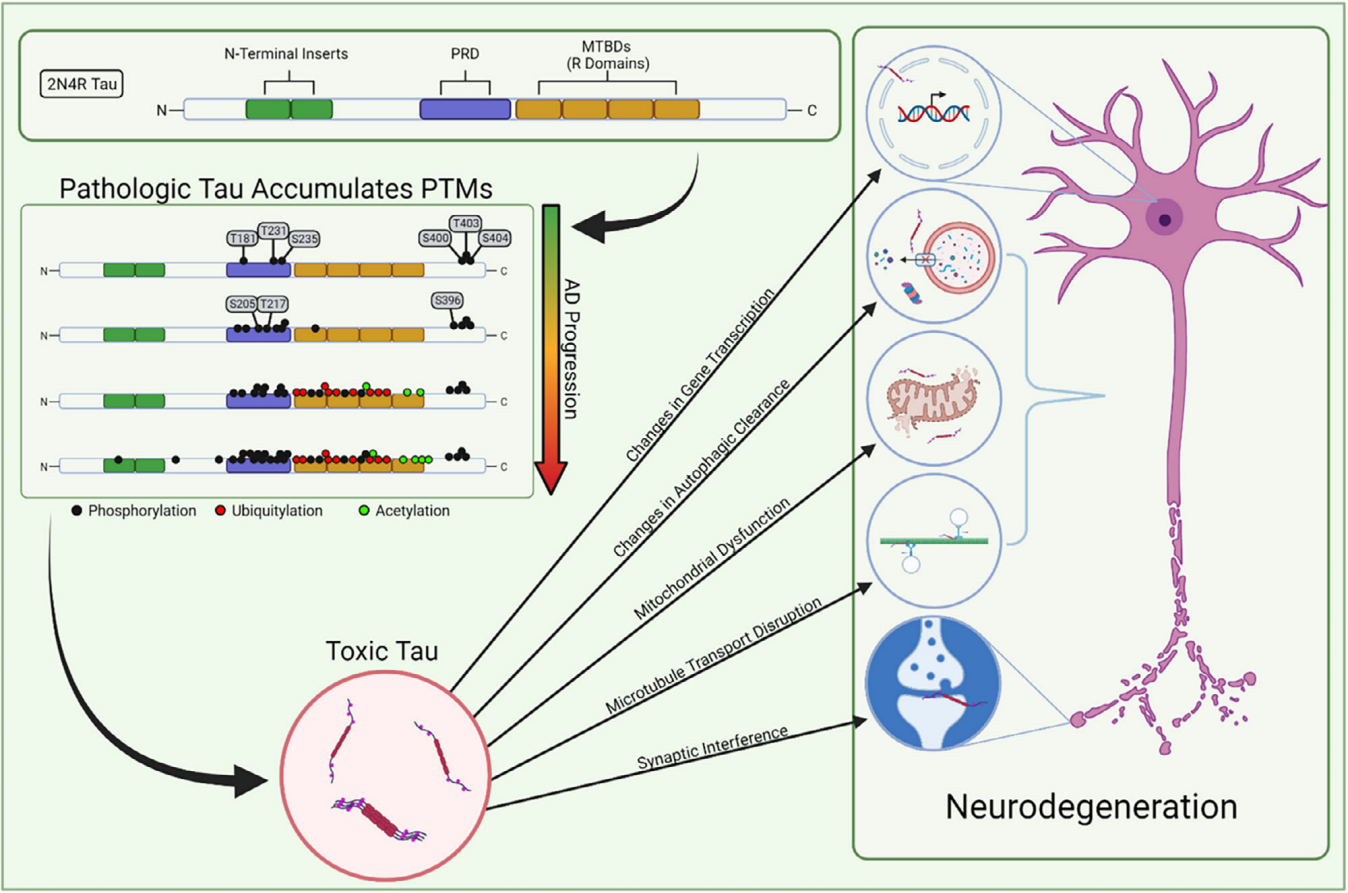
Post‐translational modifications of tau protein, such as phosphorylation, ubiquitylation, and acetylation, can contribute to the toxic tau function in AD. The accumulation of the toxic tau proteins can facilitate the following: changes in gene transcription, changes in autophagic clearance, mitochondrial dysfunction, disruption in microtubule transport, and synaptic interference. These changes contribute to neurodegeneration and neuronal death characterized in AD. Reproduced under the terms of the CC‐BY 4.0 license.^[^ ^31^ ^]^ Copyright 2021, The Author(s), published by MDPI.

Post‐translational changes, including phosphorylation, acetylation, oxidation, ubiquitination, methylation, glycosylation, and polyamination, can affect tau protein function. Polyamination is a posttranslational process. These alterations can result in enhanced aggregation of tau proteins and creation of tau tangles, both of which contribute to the pathogenesis of AD (Figure 4).^[^ ^28^, ^29^ ^]^

Understanding the function of tau proteins and their involvement in AD is essential for the development of specific therapeutic techniques that can combat the aberrant aggregation of tau proteins and alleviate some of the debilitating symptoms of this neurodegenerative condition. The ongoing research that is being conducted in this area has promise for expanding our knowledge of AD and possibly developing novel medicines to tackle this difficult condition.

## 7 TAU MUTATIONS

Tau mutations are important factors in the development of neurodegenerative disorders and AD. The repeat/inter‐repeat region of the MAPT gene, which is responsible for coding tau proteins, is where the majority of mutations occur or are located nearby.^[^ ^26^, ^32^ ^]^ These mutations cause abnormalities in the function and behavior of tau proteins.

Tau proteins that are transcribed as a result of genetic mutations have a diminished capacity to assemble microtubules in vitro and bind to them to support their assembly.^[^ ^26^, ^33^, ^34^, ^35^, ^36^ ^]^ In addition, they have a significantly increased tendency to generate aberrant tau fibers. Mutant tau molecules have been proven to cause neuronal death and dementia by blocking tau's ability to regulate microtubule instability.^[^ ^37^, ^38^, ^39^, ^40^, ^41^ ^]^ In addition, in the brains of transgenic mice that overexpress mutant tau, as well as in the brains of humans with AD, mutant tau proteins are found to alter the functioning of nuclear pores involved in nucleocytoplasmic transport, which contributes to neurotoxicity.^[^ ^29^, ^42^ ^]^

Additional effects of mutant tau proteins on microtubule dynamics include a reduction in the average growth rate of microtubules and induction of deformation of the nucleus.^[^ ^26^, ^43^ ^]^ These altered tau proteins interfere with the role of normal tau in the assembly of tubulin into microtubules and have low microtubule‐promoting capabilities.^[^ ^44^ ^]^

Two different hypotheses of molecular mechanisms have been proposed to explain how tau tangles contribute to the etiology of AD. According to the gain‐of‐toxic function concept, tau mutations are responsible for the production and accumulation of aberrant intracellular tau fibers, which ultimately results in cell death due to the cytotoxic nature of tau fibers.^[^ ^26^, ^36^, ^45^ ^]^ On the other hand, the microtubule misregulation concept suggests that tau mutations interfere with the normal capacity of tau to regulate microtubule dynamics, which ultimately leads to cell death.^[^ ^26^, ^46^, ^47^, ^48^, ^49^ ^]^ This hypothesis was supported by the observation that tau‐related diseases are common. The microtubule misregulation paradigm is consistent with research demonstrating the significant role that stringent regulation of microtubule dynamics plays in ensuring the health and survival of cells [Figure 6].^[^ ^26^, ^50^, ^51^, ^52^, ^53^ ^]^ To create targeted therapeutics to address the underlying causes of AD and other neurodegenerative conditions, it is essential to understand the molecular mechanisms underlying tau mutations and the impact of these mutations on cellular function. Ongoing research in this area continues to elucidate the intricacies of tau biology and reveal novel therapeutic intervention pathways.

## 8 TAU PHOSPHORYLATION

Tau phosphorylation is a multifaceted phenomenon characterized by the enzymatic attachment of phosphate groups to distinct serine, threonine, and tyrosine residues within the structural framework of the tau protein.^[^ ^55^ ^]^ Aberrant phosphorylation of tau protein plays a pivotal role in the pathogenesis of AD. This pathological process ultimately culminates in the formation of tau aggregates and neurofibrillary tangles, which are closely associated with the progressive degeneration of neuronal structures and subsequent decline in cognitive function.^[^ ^56^, ^57^ ^]^

Multiple kinases have been identified as key players in the process of tau phosphorylation. These kinases can be categorized into two main groups: proline‐directed and non‐proline‐directed kinases.^[^ ^50^, ^55^, ^58^, ^59^, ^60^, ^61^, ^62^ ^]^ Examples of proline‐directed kinases involved in tau phosphorylation include glycogen synthase kinase‐3 (GSK‐3) and cyclin‐dependent kinase 5 (CDK5). Non‐proline‐directed kinases such as microtubule affinity‐regulating kinases (MARKs) and casein kinase 1 (CK1) have also been implicated in this phosphorylation process.^[^ ^56^, ^63^, ^64^, ^65^, ^66^, ^67^, ^68^, ^69^, ^70^, ^71^ ^]^ In addition to its well‐established role as a microtubule‐associated protein, tau is phosphorylated by various tyrosine kinases including Fyn, Abl, and Syk.^[^ ^36^, ^55^, ^58^, ^72^ ^]^ In contrast, it is noteworthy to consider the presence of tau dephosphorylation kinases, namely, protein phosphatase‐1, −2A, and −5 (PP1, PP2A, and PP5), which play pivotal roles in the dephosphorylation of tau by facilitating the removal of phosphate groups.^[^ ^25^, ^55^, ^70^, ^73^, ^74^, ^75^, ^76^, ^77^ ^]^

**FIGURE 5:**
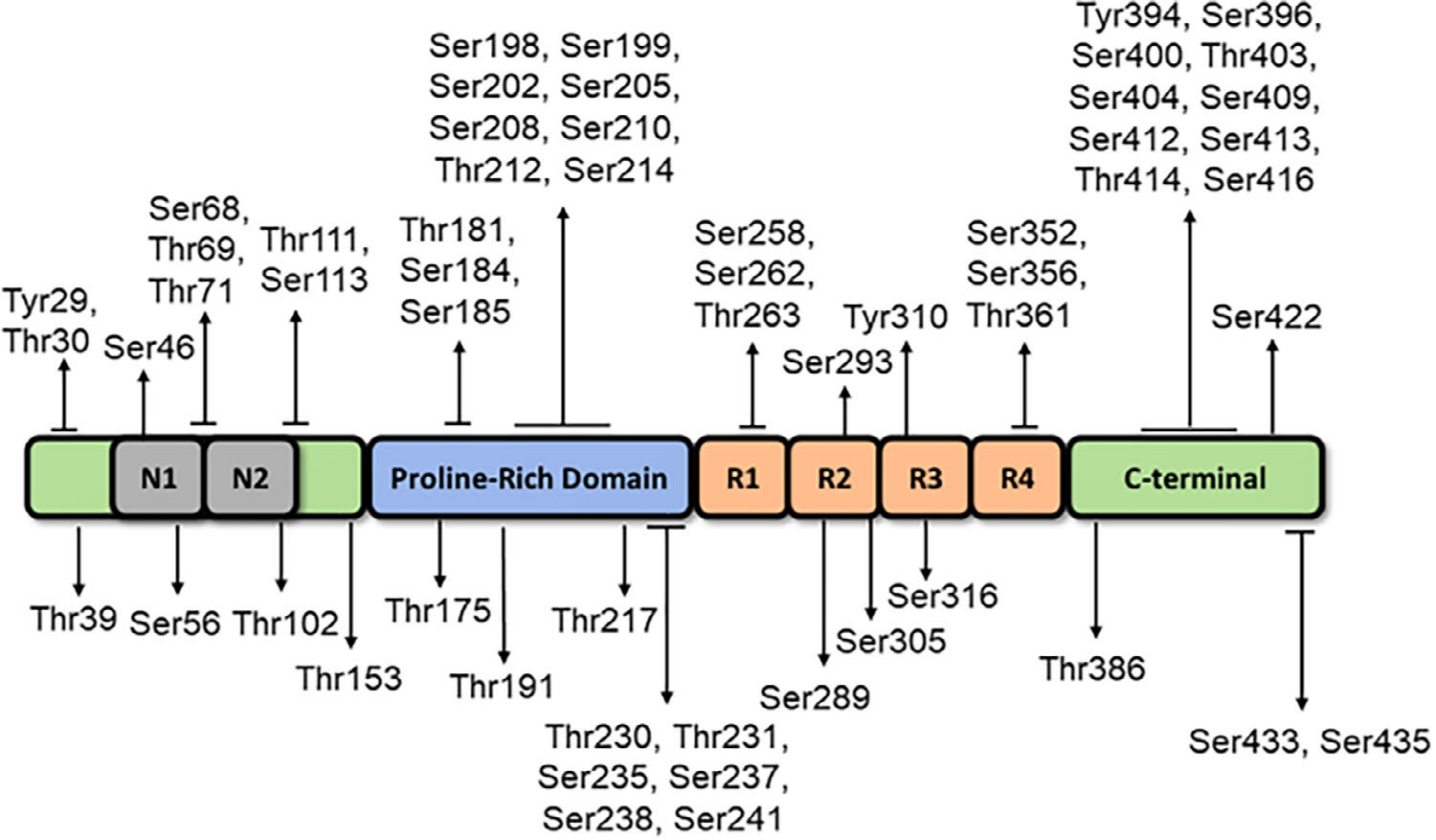
The various phosphorylation sites present on the tau proteins and their corresponding region. Reproduced under the terms of the CC‐BY 4.0 license.^[^ ^54^ ^]^ Copyright 2021, The Author(s), published by Springer Nature.

Identification of phosphorylation events at specific sites, including Thr231, Ser199, and Tyr18, has been recognized as an initial event in the progression of AD.^[^ ^56^, ^57^, ^58^, ^59^, ^62^ ^]^ As the pathological progression unfolds, there is a notable escalation in the process of phosphorylation at additional sites, namely, Ser202/Thr205 and Ser422 (See Figure 5). The accumulation of phosphorylated tau protein has been observed to have deleterious effects on microtubule dynamics, axonal transport, and synaptic function.^[^ ^55^, ^63^, ^78^, ^79^, ^80^ ^]^ These disruptions ultimately contribute to synaptic dysfunction and the subsequent cellular loss.^[^ ^81^ ^]^

**FIGURE 6:**
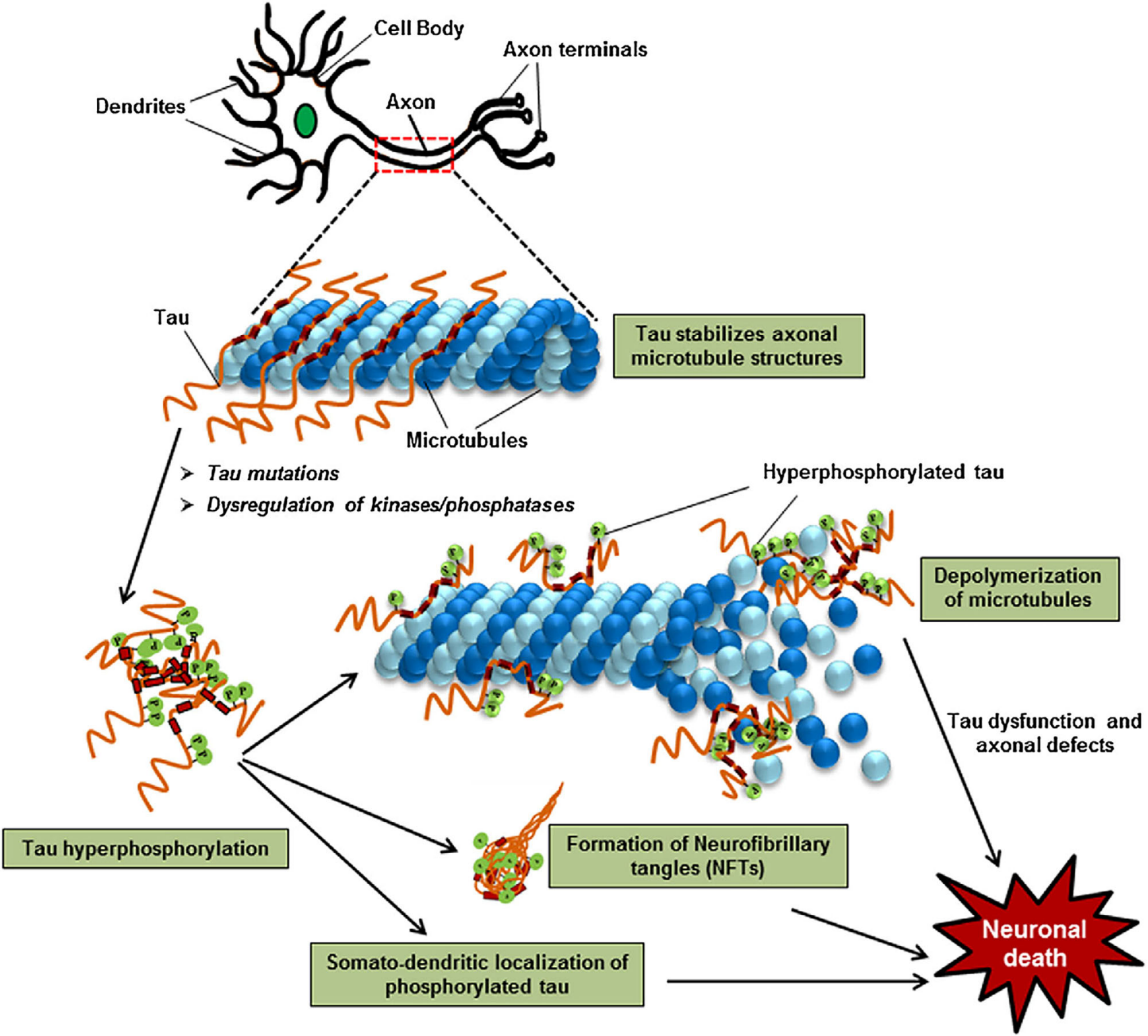
Effects of tau hyperphosphorylation on microtubules and neurons. Normal tau functions to stabilize axonal microtubule structures. Tau mutations and/or the dysregulation of kinases/phosphatases result in tau hyperphosphorylation. This eventually leads to the depolymerization of microtubules, formation of neurofibrillary tangles (NFTs), and the somato‐dendritic localization of phosphorylated tau. Lastly, tau dysfunction causes defects in the axons of neurons and causes neuronal death. Reproduced with permission.^[^ ^88^ ^]^ Copyright 2018, Indian Academy of Sciences.

The presence of extensively phosphorylated tau species has consistently been linked to synaptic dysfunction and neurodegeneration in various studies.^[^ ^44^, ^50^, ^81^ ^]^ Mitochondrial proteins can interact with external agents, resulting in disruption of mitochondrial function.^[^ ^55^, ^82^, ^83^ ^]^ This in turn results in compromised energy metabolism in neurons.^[^ ^84^ ^]^ The influence of phosphorylated tau on the distribution of mitochondria within neurons and its impact on axonal transport have been well documented in the literature.^[^ ^5^, ^55^, ^85^ ^]^

In the context of AD pathogenesis, it is widely acknowledged that tau phosphorylation plays a pivotal role in the pathogenesis of AD.^[^ ^4^, ^55^, ^84^ ^]^ Consequently, there is growing interest in investigating the therapeutic potential of targeting tau kinases or phosphatases as a means to address the underlying mechanisms of AD.^[^ ^50^, ^55^, ^86^, ^87^ ^]^ Comprehending the complexities inherent in the process of tau phosphorylation is of paramount significance in the pursuit of formulating efficacious therapeutic interventions aimed at impeding or decelerating the progression of this profound neurodegenerative disorder.

Accordingly, many progresses have been established in formulating compounds that directly inhibit tau phosphorylation. For example, the Cdk5 inhibitory peptide hinders tau hyperphosphorylation by disrupting the formation of the cdk5/p25 complex. Riscovitine, a cdk5 inhibitor, has also been found effective in reducing tau hyperphosphorylation and neurodegeneration. In vitro studies with lithium, a GSK3 inhibitor, have shown to inhibit tau phosphorylation, limit tau aggregation, mitigate neuronal degradation, and facilitate microtubule assembly. Similarly, GSK3 inhibitory compounds such as AR‐A014418 and NP‐12, have been successful in decreasing insoluble tau levels, preventing tau aggregation, downregulating tau phosphorylation, and reducing concentrations of amyloid plaques in mouse models. Furthermore, these results have been found to also mitigate neuronal death in the entorhinal cortices and hippocampus and enhance memory deficits in mouse brains. Other studies have found that the K252a compound, a serine/threonine protein kinase inhibitor, has proved useful in blocking tau hyperphosphorylation in rat brain cells. Although the aforementioned compounds have been found to be effective in limiting tau phosphorylation for applications in AD treatment, limitations such as low bioaccumulation, insignificant in vivo circulation times, and non‐specific targeting abilities have restricted the effectiveness of these therapeutic compounds. Perhaps synergizing these anti‐phosphorylation compounds with nanoparticles can overcome these limitations and offer a more effective therapeutic for hyperphosphorylation‐driven AD manifestations. The prospects of integrating nanoparticles in tau‐targeted therapies will be further explored in later sections of this paper.

## 9 TAU ACETYLATION

Acetylation of tau at lysine 280 has been recognized as a significant determinant of the molecular etiology of AD.^[^ ^89^ ^]^ Emerging scholarly investigations have provided compelling evidence that tau acetylation may precede tau hyperphosphorylation, thus potentially serving as the primary catalyst for neuronal degeneration in the early stages of AD. The presence of acetylated tau 280 was observed in all instances of early AD by Lucke‐Wold et al., thereby supporting the hypothesis that tau acetylation may occur prior to phosphorylation and exerts a pivotal influence on the process of neurodegeneration.

The process of acetylation of tau protein involves the addition of acetyl groups to specific lysine residues.^[^ ^90^ ^]^ Notably, lysine 280, which is located within the microtubule‐binding motif, has been identified as a prominent site for tau acetylation. Acetylation has been observed to induce modifications in the functionality of tau protein, thereby facilitating the progression of pathological tau aggregation and hindering its interactions with microtubules. Cohen et al. and Trzeciakiewicz et al. elucidated the detrimental effects of acetylation at K280/K281 on tau‐mediated microtubule stabilization, while concurrently promoting tau aggregation. Other studies have found that specifically, acetylation of tau proteins at the lysine 280 residue, plays a major role in propelling AD pathogenesis. Detailed studies by Irwin et al., showed that acetylated‐tau pathology played an equally significant role in AD pathogenesis as hyperphosphorylated tau. Furthermore, significant concentrations of acetylated tau were found in all stages of AD pathogenesis, with higher concentrations correlated with later stages of AD, as observed in post‐mortem AD patient's brains. Recent studies have posited that tau acetylation at lysine 280 likely contributes to AD pathogenesis by limiting tau solubility, interfering with normal microtubule assembly mechanics, and promoting the formation of tau fibrils. In the study by Caballero et al., tau acetylation was correlated with disrupting chaperone‐mediated autophagy, thus stimulating pathological tau pathways. These findings provide compelling evidence that the process of tau acetylation disturbs the regular physiological operations of tau, thereby playing a contributory role in the genesis of tau aggregates commonly observed in AD. Thus, the involvement of tau acetylation in the progression of AD has been established. Experimental investigations utilizing viral‐transduced and transgenic mouse models have provided evidence that replication of tau acetylation at distinct sites elicits AD‐like phenotypes, such as synaptic dysfunction, neuronal degeneration, and cognitive deficits. The potential therapeutic strategy of modulating the tau acetylation machinery to restrict the acetylation of residues K280 and K281 holds promise for the cessation of tau aggregation and subsequent neurodegeneration observed in AD.^[^ ^28^, ^91^, ^92^ ^]^ Deacetylases, such as HDAC6 and SIRT1, have been identified as enzymatic entities capable of catalyzing the deacetylation process of tau.^[^ ^28^, ^90^, ^93^ ^]^ The augmentation of deacetylase activity or the suppression of acetyltransferase activity holds promise for the restoration of physiological tau function and the prevention of pathological tau aggregation.

Studies have found that salsalate can prohibit tau acetylation by interfering with p300 acetyltransferase activities and compromising the structural integrity of K174 acetylation sites in transgenic mouse models. Furthermore, salsalate has significant efficacy in mitigating atrophy in hippocampal regions and preventing memory deficits. Although positive results were found in in vitro salsalate administration, its efficacy was limited in a phase 1 clinical trial. Results from the clinical trial showed that even though salsalate had optimal biocompatibility in patients, their cognitive performance was not significantly improved upon salsalate administration. Researchers hypothesized that salsalate's efficacy was likely limited by the suboptimal penetration of salsalate into the patient's brains. This limitation could be overcome by encapsulating salsalate within nanoparticulate systems, as extensive studies have shown that optimally functionalized nanoparticles often have significantly higher penetration capabilities in biological tissue, when compared to free compounds. However, to definitively establish this, further research is required.

In AD research, tau acetylation has garnered considerable attention as a notable factor in the development and progression of the disease.^[^ ^28^, ^89^, ^90^, ^92^ ^]^ Consequently, directing interventions towards the modulation of acetylation machinery presents a promising avenue for therapeutic exploration, with the potential to revolutionize the treatment of AD and ameliorate its neurodegenerative consequences. Further investigation is warranted to attain a comprehensive understanding of the intricacies of tau acetylation and its plausible therapeutic ramifications.

## 10 TAU OXIDATION

Tau oxidation plays a pivotal role in the etiology of AD.^[^ ^94^, ^95^ ^]^ Numerous experimental models and investigations conducted in the human brain have demonstrated the pivotal involvement of oxidative stress in the process of neurodegeneration, particularly in the early stages of AD.

Mitochondrial dysfunction has been closely linked with the occurrence of oxidative stress, thereby serving as an incipient stimulus for the generation of amyloid‐beta, a pivotal protein implicated in the pathogenesis of AD.^[^ ^96^, ^97^, ^98^ ^]^ Accumulation of amyloid‐beta has been observed to accelerate oxidative stress and exacerbate mitochondrial dysfunction.^[^ ^98^, ^99^, ^100^ ^]^

Oxidative stress has been identified as a significant factor in the pathogenesis of neurofibrillary tangles and aberrant accumulation of hyperphosphorylated tau protein, which are prevalent in the cerebral cortex of individuals with AD. The identification of fatty acid oxidative products has established a direct association between the mechanisms underlying oxidative stress and the genesis of neurofibrillary tangles.^[^ ^98^, ^101^ ^]^

In tandem with other contributing factors such as the presence of okadaic acid, oxidative stress has been observed to result in tau hyperphosphorylation.^[^ ^98^, ^102^ ^]^ The hyperphosphorylation of tau protein is hypothesized to arise from the interplay between oxidative stress and tau protein kinases and phosphatases, namely GSK‐3β and PP2A. Multiple studies have demonstrated that glycogen synthase kinase‐3 beta (GSK‐3β) activity is enhanced in response to oxidative stress.

The intricate relationship between oxidative stress and tau pathology is multifaceted and has significant importance in the development and progression of AD. Oxidative stress perturbs the regular functioning of cellular mechanisms, thereby giving rise to generating harmful entities such as reactive oxygen species (ROS). ROS, in turn, inflict damage to neurons and actively participate in the initiation and progression of AD. When subjected to hyperphosphorylation in the presence of oxidative stress, the tau protein exhibits alterations in its conformational structure, thereby facilitating its aggregation into neurofibrillary tangles. This phenomenon exacerbates neurodegeneration.

Antioxidant defenses for applications in AD treatment have been explored. Antioxidant defenses vary among preventative measures, radical‐scavengers, and de novo and repair enzymes. Preventative antioxidant measures include metal‐chelating proteins, superoxide dismutase, and glutathione peroxidase. Radical scavenging antioxidants include vitamins C and E. Repair and de novo enzymes include DNA repair enzymes, lipase, and protease. Currently, the metal chelating agent, clioquinol, vitamin C, and vitamin E are undergoing extensive clinical trials and epidemiological studies to determine if the administration of these compounds can ameliorate the progression of AD. Furthermore, it is plausible to test the integrative treatment of the aforementioned antioxidant compounds with nanoparticles to investigate whether their efficacy can be enhanced with the benefits (e.g., increased blood‐brain barrier penetration, higher bioaccumulation) of nanoparticle delivery systems.

The exploration of therapeutic strategies for AD has led researchers to focus on the intricate relationship between oxidative stress and the tau phosphorylation pathways. By targeting oxidative stress and its impact on these pathways, there is a promising opportunity to develop effective AD treatments. The amelioration of tau pathology and neurodegeneration in AD can be achieved by mitigating oxidative stress and restoring cellular antioxidant defenses. Further investigation is warranted to comprehensively understand the intricate mechanisms at play and cultivate efficacious therapeutic interventions that specifically address the process of tau oxidation in AD.

## 11 APPLICATION OF NANOPARTICLES (NPS) FOR TARGETED THERAPY OF ALZHEIMER'S DISEASE

AD is an incapacitating neurodegenerative condition characterized by the aggregation of pathogenic tau proteins, resulting in cognitive deterioration and amnesia. Existing pharmacological interventions endorsed by the Food and Drug Administration (FDA) address the management of symptoms associated with AD, failing to impede the advancement of pathological conditions.^[^ ^1^] This underscores the need for the development of novel therapeutic approaches that not only exhibit enhanced efficacy, but also possess the potential to achieve a complete cure. The remainder of this paper will focus on the research surrounding the development of the novel proteotoxic‐tau targeting therapeutics. In recent years, a growing body of evidence has suggested that experimental drugs and intervention systems have significant potential for therapeutic applications. Nevertheless, the broad implementation of these novel approaches is impeded by certain inherent limitations, namely their modest toxicological profile and restricted ability to traverse the blood‐brain barrier (BBB).^[^ ^103^ ^]^ In response to these obstacles, the utilization of nanoparticles (NPs) in targeted drug delivery systems has emerged as a highly encouraging and viable strategy.

Nanoparticles present a multitude of advantageous attributes, including heightened drug effectiveness, enhanced compatibility with biological systems, elevated availability within the biological milieu, and precise administration to specific cerebral regions affected by AD.^[^ ^12^ ^]^ Successful blood‐brain barrier (BBB) penetration and subsequent controlled drug release, along with the attainment of higher drug concentrations at AD sites, can be facilitated through the process of functionalizing ligands onto the surfaces of NPs. This comprehensive review provides an overview of diverse categories of NPs, including polymeric NPs, liposomes, metallic NPs, magnetic NPs, silica NPs, and carbon NPs (See Figure 8). The primary focus of this review is to elucidate the applications of these NPs in the investigation and targeted intervention of pathogenic tau in AD.

**FIGURE 7:**
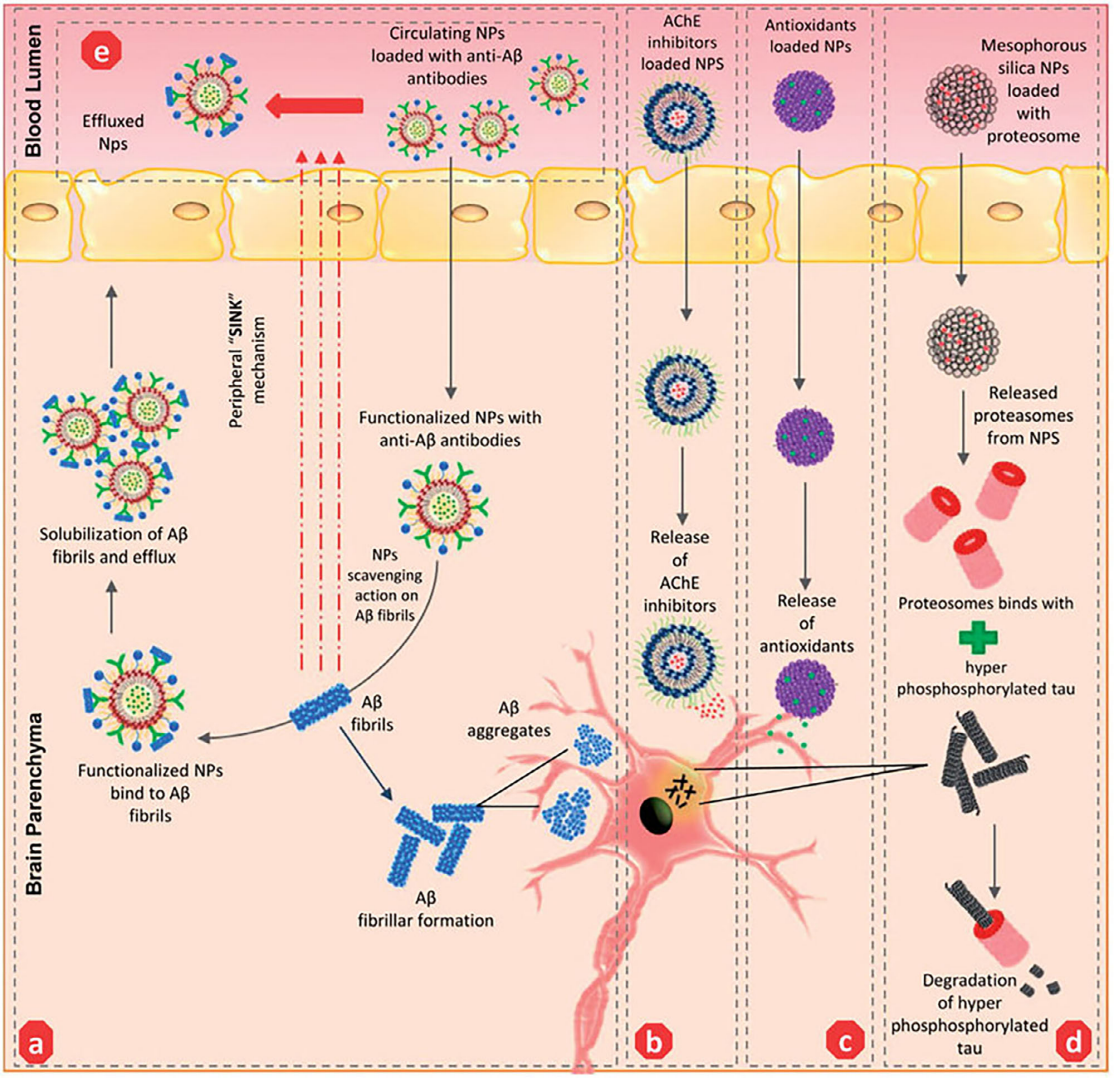
General configuration of nanoparticles conjugated for treatment of AD. NPs can be loaded with a variety of AD therapeutics, such as anti‐amyloid‐beta antibodies, acetylcholinesterase (AChE) inhibitors, antioxidants, and proteosomes to degrade hyperphosphorylated tau. Different materials can be utilized for the shell of NPs. The NPs are also functionalized so that they can be transported from the blood lumen into the brain parenchyma. Reproduced under the terms of the CC‐BY 4.0 license^[^ ^104^ ^]^ Copyright 2018, The Author(s), Published by Informa UK Limited.

**FIGURE 8:**
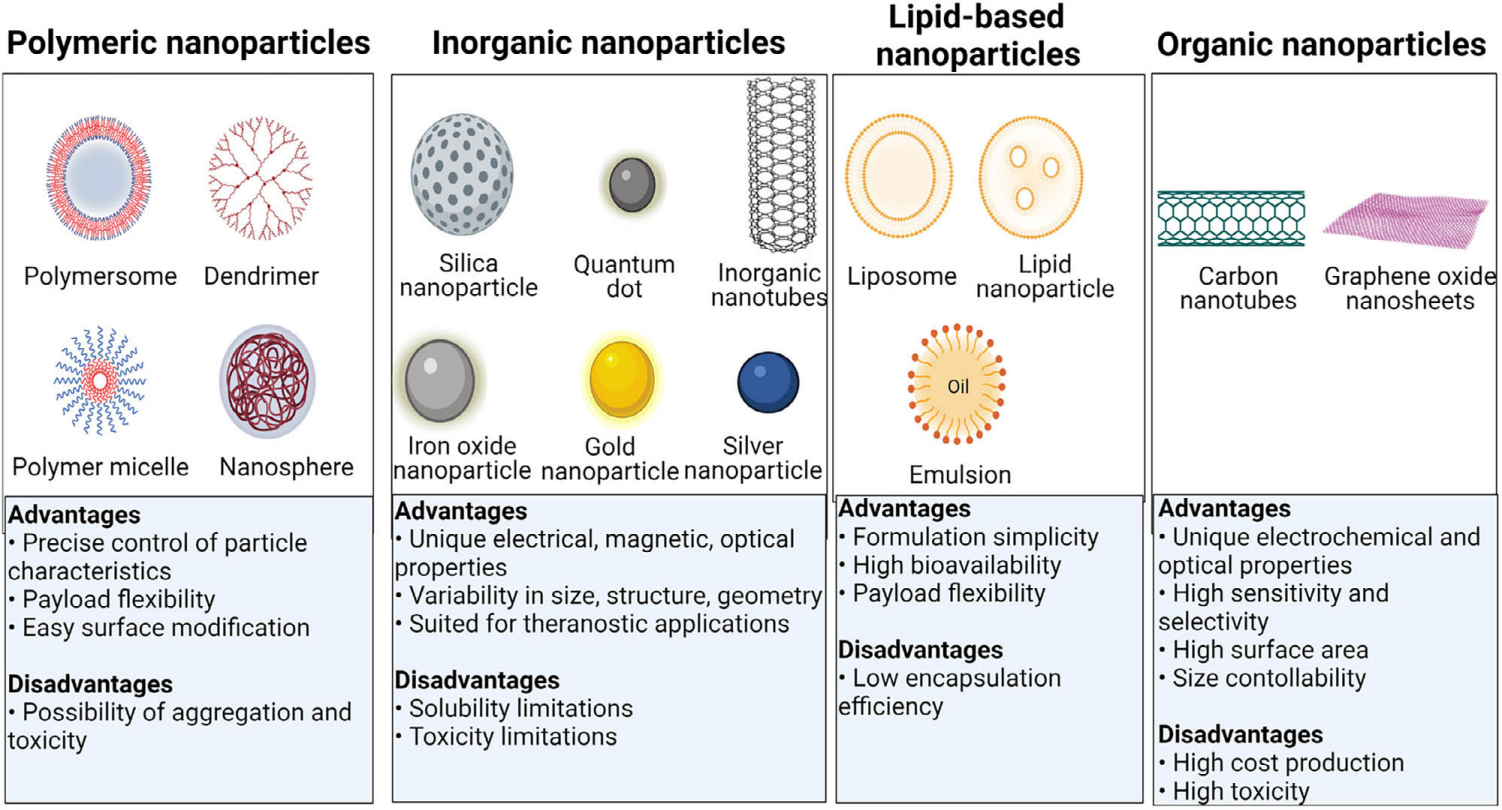
Schematic of the different nanoparticles discussed in the next sections. The advantages and disadvantages of each nanoparticle classification is summarized above. Reproduced with permission.^[^ ^105^ ^]^ Copyright 2022, The Author(s), under exclusive license to American College of Neuropsychopharmacology.

The utilization of nanotechnology in the context of targeted therapy for AD exhibits considerable potential and may constitute a paradigm shift in the pursuit of curative interventions. Nevertheless, it is imperative to conduct additional research and clinical trials to ascertain the safety and effectiveness of interventions based on NPs prior to their extensive use in the management of AD. Ongoing progress in the field of nanotechnology presents an auspicious opportunity to fundamentally transform the treatment of AD and enhance the quality of life for the substantial population afflicted by this incapacitating condition.

## 12 POLYMERIC NANOPARTICLES (NPS)

Polymeric nanoparticles are particles of small size that have gained attention in recent years for their applications as drug delivery systems. Polymersomes, dendrimers, micelles, and nanospheres are all examples of polymeric nanoparticles and can be schematically visualized in Figure 8. Drug compounds can either be encapsulated inside the polymeric nanoparticles or adsorbed onto the polymeric core. The relatively small size of polymeric NPs makes them an attractive choice, especially in regard to BBB permeability. Recent research has been focused on developing biodegradable polymer nanoparticles. This formulation offers advantages such as the degradation of the polymer shell to release the drug compounds encapsulated within the core into the biological environment. Furthermore, other advantages of polymeric nanoparticles include their relatively easy ability to control precision of particle properties, payload flexibility, and simple surface functionalization. One notable disadvantage of polymeric nanoparticles is their susceptibility to aggregate and facilitate cytotoxicity. Despite this, polymeric nanoparticles (NPs) have exhibited considerable potential for drug delivery, particularly in the context of therapeutic interventions for AD. The aforementioned materials possess notable attributes such as elevated biocompatibility, augmented biodegradability, and regulated release characteristics.^[^ ^106^ ^]^ Numerous investigations have examined the utilization of polymeric nanoparticles (NPs) as a means of delivering therapeutic agents, including curcumin, amyloid‐beta generation inhibitors, and BACE 1 antisense genes, for the treatment of AD.

Curcumin, a compound renowned for its notable antioxidant and anti‐inflammatory properties, has been incorporated into chitosan‐coated polylactic‐co‐glycolic acid (PLGA) NPs with the objective of enhancing its solubility and stability.^[^ ^107^, ^108^ ^]^ The aforementioned nanoparticle formulations exhibited superior biocompatibility, regulated drug release, and diminished cytotoxicity compared to unbound curcumin. In vivo experiments involving curcumin‐loaded NPs have demonstrated enhanced delivery efficiency and improved distribution within the brain. This has resulted in a reduction in oxidative stress levels and the emergence of potential therapeutic advantages for AD.

As an alternative methodology, the research team devised neuron tau‐targeting biomimetic NPs by encapsulating poly(lactic‐*co*‐glycolic acid) (PLGA) NPs with red blood cell (RBC) membranes, followed by functionalization with tau‐PET tracers.^[^ ^109^ ^]^ The aforementioned NP entities have demonstrated notable efficacy in traversing the blood‐brain barrier (BBB) and exhibit a strong affinity for hyperphosphorylated tau. Consequently, this interaction leads to a decrease in levels of phosphorylated tau and subsequent neuronal cell death, as observed in both controlled laboratory settings (in vitro) and living organisms (in vivo). Administration of NPs to transgenic mouse models of AD resulted in notable amelioration of memory deficits and alleviation of AD‐related symptoms.

In addition, the utilization of NPs encapsulating an amyloid‐beta generation inhibitor known as S1, along with curcumin, and conjugated with brain‐targeting peptides, resulted in significant reductions in cytokine production, restoration of antioxidant activity, and a decrease in oxidative stress levels in mouse models of AD.^[^ ^110^ ^]^ The aforementioned nucleotide polymorphism NPs have exhibited promising capabilities in ameliorating the progression of AD through precise modulation of distinct molecular pathways.

In addition, a comprehensive delivery system encompassing genetic material and peptides encapsulated within PEGylated dendrigraft poly‐l‐lysine nanoparticles was developed.^[^ ^111^ ^]^ The primary objective of this system was to downregulate BACE, BACE 1, an essential enzyme implicated in the generation of amyloid‐beta plaques. The successful delivery of the BACE 1 antisense gene and tau‐related fibril inhibitor peptide by nanoparticle (NP) carriers resulted in a notable decrease in BACE positive signals and a reduction in p‐tau positive signals in mice with AD.

These studies underscore the considerable promise of polymeric NPs in their capacity to serve as efficacious vehicles for drug delivery in the context of AD therapy. Polymeric nanoparticles (NPs) exhibit great potential in the realm of AD therapy because of their ability to enhance the delivery and targeting of therapeutic agents. Nevertheless, it is imperative to conduct additional investigations and rigorous clinical trials to comprehensively evaluate the safety and effectiveness of these interventions before their widespread adoption [see Figure 7 and Table 2].

**TABLE 2:** Summary of polymeric nanoparticles for tau‐targeting applications in Alzheimer's disease.

Nanoparticle type	Objective	Size	Dose administered	Experimental system	Notable results	Reference
Chitosan‐coated PLGA nanoparticles	Determine whether PLGA nanoparticles improve the therapeutic efficacy of curcumin through stimulation of its antioxidant and anti‐inflammatory properties	Not specified	2 mg kg^−1^	In vitro and in vivo with SH‐SY5Y and BV‐2 cells	The synthesized nanoparticle decreased the anti‐inflammatory biomarkers, IL‐6 and TNF‐a, by 40% and 70% respectively. Additionally, curcumin biocompatibility, penetration, and bioaccumulation were greater when chitosan‐coated PLGA nanoparticles were administered.	[107, 108]
Hydroxypropyl‐β‐cyclodextrin‐encapsulated curcumin nanoparticles	Develop a nanoparticulate system for enhanced delivery and penetration of curcumin in brain tissue	Not specified	2 mg kg^−1^	In vitro and in vivo with SH‐SY5Y and BV‐2 cells	The hydroxypropyl‐β‐cyclodextrin encapsulated curcumin nanoparticles had enhanced biostability when compared to curcumin‐loaded chitosan‐coated PLGA nanoparticles. Furthermore, these nanoparticles had significantly higher bioaccumulation in brain cells than curcumin‐loaded chitosan‐coated PLGA nanoparticles and free curcumin. The synthesized nanoparticles also had comparable anti‐inflammatory effects to curcumin‐loaded chitosan‐coated PLGA nanoparticles.	[107, 108]
PLGA nanoparticles functionalized with red blood cell (RBC) membranes and tau‐PET tracers	Synthesize a nanoparticulate system that demonstrates enhanced permeation through the BBB to effectively target hyperphosphorylated tau proteins	Not mentioned	Not mentioned	In vitro and in vivo with transgenic mouse models	The PLGA nanoparticles functionalized with RBC membranes and tau‐PET tracers demonstrated enhanced efficacy in penetrating the BBB and showed a significant affinity for hyperphosphorylated tau. Further in vitro and in vivo studies showed that the synthesized NPs decreased phosphorylated tau levels, neuronal cell death, and memory deficits.	[112]
PLGA nanoparticles loaded with amyloid‐β generation inhibitor (S1) and curcumin, and conjugated with transferrin receptor targeting CRT peptide	Implement a nanoparticle delivery system that can overcome the following limitations posed by free drug delivery for treatment of AD: ineffective BBB penetration, limited bioavailability, and low circulation times	128.6–139.8 nm	0.5 mg kg^−1^ and 2 mg kg^−1^	In vitro and in vivo studies with SH‐SY5Y human neuroblastoma cells, BV2 mouse microglial cells, and bEnd.3 mouse‐derived brain capillary endothelial cells.	Y‐maze and object recognition tests showed that the synthesized NPs significantly improved spatial memory in AD transgenic mice. Furthermore, these NPs reduced amyloid‐β levels, reactive oxygen species (ROS), and anti‐inflammatory biomarkers (i.e., TNF‐α and IL‐6). Along with these benefits, the S1 and curcumin loaded PLGA NPs functionalized with CRT restored optimal superoxide dismutase activities and facilitated synaptogenesis in AD mouse brains.	[110]
PEGylated dendrigraft poly‐l‐lysine nanoparticles	Develop an interventional nanoparticulate system to downregulate BACE 1, an essential enzyme implicated in the formation of amyloid‐β plaques and tau‐related fibrils	Not specified	50 µg	In vitro: brain capillary endothelial cells and SH‐SY5Y cells In vivo: APPswe and PSEN1dEP transgenic mice	Successful delivery of the antisense siRNA BACE and tau‐related fibril inhibitor peptide was achieved. Furthermore, the PEGylated dendrigraft poly‐l‐lysine nanoparticles significantly decreased positive BACE 1 signals and phosphorylated‐tau levels in AD mouse models.	[113]

## 13 LIPID NANOPARTICLES

Lipid nanoparticles are often composed of single‐layer or multiple‐layers of phospholipids. Furthermore, the amphipathic nature of lipid nanoparticles allows them to offer unique drug‐encapsulation capabilities. Hydrophilic drug compounds can be embedded within the lipid nanoparticles’ aqueous compartments, while hydrophobic drug compounds can be encapsulated with their membranes. The biological similarity of lipid nanoparticles and cellular membranes allow lipid nanoparticles to penetrate and accumulate in biological tissue with enhanced efficacy, compared to other nanoparticle counterparts. Furthermore, lipid nanoparticles’ formulation simplicity, high bioavailability, and payload flexibility contribute to their widespread use in biomedical research. One limitation of lipid nanoparticles is their low encapsulation efficiency. However, their ability to prevent the loaded drug compound from degradation make them an attractive choice to employ for therapeutic applications. Specifically, lipid nanoparticles, including liposomes as a prominent example, have shown considerable promise as efficacious vehicles for the targeted administration of therapeutic agents in the context of AD intervention.^[^ ^111^ ^]^ Numerous studies have been conducted to examine the utilization of lipid nanoparticles as a vehicle for administering therapeutic agents and impeding pivotal pathological mechanisms, such as tau aggregation, linked to AD.^[^ ^114^, ^115^ ^]^

Ross et al. developed a novel approach for creating liposomes with multiple functions.^[^ ^114^ ^]^ These liposomes were specifically designed to encapsulate curcumin, which is a bioactive compound with potential therapeutic applications. Additionally, liposomes were modified with nerve growth factor (NGF), cardiolipin (CL), and wheat germ agglutinin (WGA) to enhance their functionality. Curcumin is widely recognized for its notable attributes in the mitigation of inflammation and inhibition of tau phosphorylation.^[^ ^114^, ^116^ ^]^ The neurotrophic factor known as NGF mitigates neuronal apoptosis, thereby potentially promoting neuronal survival. In contrast, CL enhances the affinity of liposomes towards neurons in close proximity to amyloid‐beta accumulations. The facilitation of blood‐brain barrier (BBB) penetration is a notable effect of WGA compounds. The synthesized liposomes significantly downregulated the expression of phosphorylated p38 proteins, phosphorylated tau protein levels, and phosphorylated c‐Jun N‐terminal kinase. Furthermore, the liposomes demonstrated excellent efficacy in improving BBB penetration, decreasing amyloid‐β accumulation, stimulating axon genesis, and preventing neurodegeneration. Thus, in vitro investigations have demonstrated a notable decrease in the accumulation of amyloid‐beta and tau proteins, thereby indicating potential therapeutic implications for AD.

Vakilinezhad et al. undertook the task of formulated solid lipid nanoparticles (SLNs) specifically engineered to encapsulate nicotinamide, an inhibitor of histone deacetylase (HDAC).^[^ ^111^ ^]^ The rationale behind this design was to investigate the potential of nicotinamide loaded SLNs to mitigate tau hyperphosphorylation, a pathological process associated with cognitive impairment. We hypothesized that the delivery of nicotinamide via SLNs could enhance its therapeutic efficacy in improving cognitive function. Researchers have successfully enhanced brain delivery, mitigated neurotoxicity, and enhanced cognitive outcomes in animal models of AD by modifying solid lipid nanoparticles (SLNs) with polysorbate 80, phosphatidylserine (PS), or phosphatidic acid (PA). In vitro studies confirmed the biocompatibility of the phosphatidyl‐serine and phosphatidic acid functionalized nicotinamide‐loaded SLNs. Biodistribution studies showed that the synthesized SLNs improved brain delivery of nicotinamide across the BBB. Furthermore, Morris water maze tests and memory examinations supported that the synthesized SLNs enhanced cognition, cultivated normal neuronal cell functionalities, and inhibited tau hyperphosphorylation. The utilization of PS‐SLNs demonstrated notable efficacy in impeding the progression of AD through the reduction of tau hyperphosphorylation.

Song et al. formulated lipoprotein‐based nanoparticles that were strategically functionalized with ApoE3.^[^ ^117^ ^]^ The primary objective of this innovative approach was to augment the permeability of the blood‐brain barrier (BBB) and subsequently ameliorate memory impairments in mice with AD. Nanoparticles exhibit a propensity for binding to amyloid‐beta, thereby facilitating its degradation through the concerted efforts of microglia and astroglia. Additionally, they play a crucial role in promoting hepatic elimination of amyloid‐beta, thereby mitigating amyloid deposition and alleviating neurological alterations. Detailed studies indicated that the synthesized nanostructures could travel across the BBB, stimulate amyloid‐β and tau degradation, facilitate microgliosis, and restore memory capabilities in AD mouse models. Thus, the nanoparticles under investigation exhibit promising attributes that render them viable candidates for safe and biocompatible therapeutic interventions.

Hu et al. employed the technique of encapsulating tannic acid within liposomal nanoparticles to impede the process of tau aggregation.^[^ ^118^ ^]^ The interaction between tannic acid and tau peptides impedes the folding process of the latter, thereby inhibiting the formation of intricate conformations and subsequently mitigating the aggregation of tau fibrils. The ability of tannic acid liposomes to traverse the blood‐brain barrier (BBB) implies their prospective utility as therapeutic interventions for AD.

In the realm of AD therapy, Lipid nanoparticles have emerged as a promising avenue for targeted drug delivery and inhibitors of tau aggregation in AD therapy. The aforementioned studies underscore the potential of these nanoparticles in impeding crucial pathological mechanisms and fostering cognitive enhancement in AD models [See Table 3].

**TABLE 3:** Summary of tau‐targeting liposomal nanoparticles for applications in AD treatment.

Nanoparticle type	Objective	Size	Dose administered	Experimental system	Notable results	References
Curcumin‐loaded liposomes functionalized with NGF, CL, and WGA	Synthesize a more effective curcumin‐delivery system to decrease phosphorylated tau levels and alleviate AD symptoms	130–145 nm	0.8–1.0 mg mL^−1^	In vitro: SK‐N‐MC cells In vivo: Wistar rats	The synthesized liposomes significantly downregulated the expression of phosphorylated p38 proteins, phosphorylated tau protein levels, and phosphorylated c‐Jun N‐terminal kinase. Furthermore, the liposomes demonstrated excellent efficacy in improving BBB penetration, decreasing amyloid‐β accumulation, and preventing neurodegeneration	[114]
Solid lipid nanoparticles (SLN) encapsulated with nicotinamide, a histone deacetylase inhibitor	Design an interventional system to ameliorate AD symptoms by inhibiting tau hyperphosphorylation and determine which functionalization (polysorbate 80, phosphatidylserine, phosphatidic acid) was most effective	Polysorbate‐80 functionalized nicotinamide‐loaded SLNs: 112 nm Phosphatidylserine‐functionalized nicotinamide‐loaded SLNs: 124 nm Phosphatidic acid functionalized nicotinamide loaded SLNs: 137 nm	15, 30, 60 mg kg^−1^	In vitro: SH‐SY5Y cells	In vitro studies confirmed the biocompatibility of the phosphatidyl‐serine and phosphatidic acid functionalized nicotinamide‐loaded SLNs. Biodistribution studies showed that the synthesized SLNs improved brain delivery of nicotinamide. Furthermore, Morriswater maze tests and memory examinations supported that the synthesized SLNs enhanced cognition, cultivated normal neuronal cell functionalities, and inhibited tau hyperphosphorylation	[111]
Apolipoprotein E3 (apoE3) and high‐density lipoprotein (HDL) functionalized liposomes	Create a nanostructure that is capable of effectively permeating the BBB and enhancing amyloid‐β and tau clearance	Not specified	5 mg kg^−1^	In vivo: SAMP8 mice	Detailed studies indicated that the synthesized nanostructures could travel across the BBB, stimulate amyloid‐β and tau degradation, facilitate microgliosis, reverse AD‐induced neurologic changes, and restore memory capabilities in AD mouse models.	[117]
Liposomal nanoparticles encapsulated with tannic acid and coated with Tween‐80	Synthesize a liposomal nanoparticulate system focused on inhibiting tau aggregation for applications in AD treatment	Not specified	Not specified	In vitro: SK‐N‐SH neuroblastoma cell line and bEnd.3 mouse‐derived microvascular brain endothelial cells	Extensive in vitro studies indicated that the synthesized nanoparticles could effectively traverse the BBB and decrease tau aggregation induced tau peptide R3 fibrils	

## 14 GOLD NANOPARTICLES (AU NPS)

Gold nanoparticles (Au NPs) have garnered considerable interest in the fields of biotechnology, biomedicine, and radiation medicine, primarily because of their distinctive attributes, including size‐dependent optical properties and minimal cytotoxicity.^[^ ^113^ ^]^ Au NPs are favorable for biomedical applications due to their unique, electrical, magnetic, and optical properties that can be adapted to suit a myriad of functions. Furthermore, their variable geometry, size, and structure render them an attractive choice for diagnostic and therapeutic applications. Additionally, their tunable characteristics and modifiable diameters allow Au NPs to traverse the Blood Brain Barrier (BBB) with relative ease. Within the realm of AD, it is worth noting that Au NPs present themselves as a potentially fruitful avenue for exploration in terms of both diagnostic and therapeutic applications. Au NPs have been employed as colorimetric sensors and metal ion detectors to investigate the pathogenesis of AD.

The research conducted by Neely et al. developed AuNPs conjugated with anti‐tau antibodies.^[^ ^113^, ^119^ ^]^ This conjugation allowed for the detection of tau protein concentration by leveraging the interaction between the antibodies and tau protein antigen. Utilization of this particular methodology facilitated the identification of exceedingly minute quantities of amyloid‐beta and tau proteins in cerebrospinal fluid, thereby establishing the potential of Au NPs as a viable diagnostic instrument for AD.

Similarly, Zhang et al. developed a formulation of AuNPs surface‐conjugated with a polyA aptamer (PAapt).^[^ ^120^ ^]^ The objective of their study was to enable simultaneous detection of amyloid‐beta (1‐42) oligomers and tau protein levels. The remarkable specificity exhibited by PAapt AuNPs in their ability to selectively identify tau proteins and amyloid‐beta (1‐42) oligomers, while remaining unaffected by the presence of non‐target proteins, renders them highly promising candidates for the development of efficient clinical diagnostic tools aimed at detecting multiple protein biomarkers associated with AD.

A series of investigations conducted by Vimal et al. elucidated the therapeutic advantages associated with the use of Au‐PEG nanoparticles in the context of AD.^[^ ^121^ ^]^ The administration of nanogold polyethylene glycol (PEG) conjugate resulted in notable enhancements in cognitive function among transgenic tau P301L mutant mice, as evidenced by a reduction in the levels of phosphorylated tau and total tau. Au‐PEG NPs have demonstrated notable inhibitory effects on amyloidosis in ex vivo studies involving patients with AD. These findings indicate that Au‐PEG NPs hold promise as a viable treatment for AD.

In addition, a study conducted by Ghalandari et al. involved the synthesis of folic acid‐functionalized Au nanoparticles as well as gold iron oxide (Fe_3_O_4_) core‐shell nanoparticles.^[^ ^112^, ^122^ ^]^ Notably, both types of nanoparticles exhibited notable affinity for tubulin and tau proteins. Functionalized Au NPs exhibit considerable potential for the development of targeted drug delivery systems for the treatment of AD.

The utilization of Au NPs in AD research is highly advantageous because of their distinctive characteristics and compatibility with biological systems. The provision of opportunities for early diagnosis and potential therapeutic interventions represents a significant advancement in the field as it introduces novel avenues for effectively addressing the challenges presented by AD. However, it is imperative to conduct additional research and clinical studies to comprehensively understand and exploit the potential of Au NPs in the management of AD [See Table 4].

**TABLE 4:** Summary of tau‐targeting gold nanoparticles for applications in AD diagnosis and treatment.

Nanoparticle type	Objective	Size	Dose administered	Experimental system	Notable results	References
Gold nanoparticles conjugated with anti‐tau antibodies	Synthesize a highly sensitive AD diagnostic by monitoring tau protein concentrations	Not specified	Not specified	Not applicable	The anti‐tau antibody conjugated gold nanoparticles can detect significantly low tau protein concentrations of 1 pg mL^−1^	[113, 119]
Gold nanoparticles surface‐conjugated with polyA aptamer	Synthesize a highly sensitive AD diagnostic by monitoring amyloid‐β and tau protein concentrations	Not specified	Not specified	Not applicable	The polyA aptamer conjugated gold nanoparticles show promising abilities to function as an AD diagnostic by monitoring amyloid‐β and tau protein levels	[120]
Nanogold polyethylene glycol (PEG) conjugate	Develop an interventional system that ameliorates AD symptoms by restoring normal tau protein mechanics and inhibiting amyloidosis	180–260 nm	Not specified	In vivo: transgenic tau P301L mice Ex vivo: Monkey and human serum samples	Nanogold PEG conjugates can diminish total tau and phosphorylated tau levels in in vivo mouse models. Additionally, ex vivo studies indicated the Gold‐PEG nanoparticles repaired abnormal tau protein mechanics and contributed to the stabilization of proteotoxic tau	[121]
Folic‐acid functionalized gold nanoparticles	Implement a nanoparticulate system that can treat AD by stabilizing microtubules and enhancing tau clearance	Not specified	Not specified	Not specified	The folic‐acid functionalized gold nanoparticles exhibited notable affinity for stabilizing tubulin proteins and clearing tau proteins	[122]
Gold iron oxide core–shell nanoparticles	Implement a nanoparticulate system that can treat AD by stabilizing microtubules and enhancing tau clearance	Not specified	Not specified	Not specified	The folic‐acid functionalized gold nanoparticles exhibited notable affinity for stabilizing tubulin proteins and clearing tau proteins	[122]

## 15 OTHER METALLIC NANOPARTICLES

Promising results have been observed in the utilization of metallic nanoparticles, including iron and titanium nanoparticles, for diminishing tau protein concentrations and impeding tau aggregation in therapeutic interventions targeting AD. Metallic nanoparticles, such as iron and titanium nanoparticles, have unique electrical, magnetic, and optical properties that can be modified to facilitate enhanced therapeutic efficacy. The limited solubility and relative toxicity of metallic nanoparticles are two main disadvantages of implementing metallic nanoparticles in biomedical therapeutic applications.

The study conducted by Sonawane et al. studied the development and utilization of protein‐capped nanoparticles to effectively impede tau aggregation in AD.^[^ ^123^ ^]^ Researchers discovered that the utilization of protein‐capped iron oxide and cadmium sulfide nanoparticles exhibited a significant inhibitory effect on the process of tau polymerization, thereby effectively impeding the aggregation of tau proteins. The experimental findings indicated that protein‐capped nanoparticles exhibited notable efficacy in suppressing tau aggregation. Specifically, the protein‐capped cadmium sulfide nanoparticles exhibited a remarkable 63% inhibition rate, whereas the protein‐capped iron oxide nanoparticles demonstrated a significant inhibition rate of 49%. Moreover, it has been observed that iron oxide nanoparticles synthesized through biological means can impede the process of tau aggregation. This was achieved through the adsorption of intermediate tau aggregates onto the surface of these nanoparticles, thereby hindering any subsequent fibrillation. The experimental results indicate that the nanoparticles capped with proteins exhibited a notable decrease in their cytotoxic effects and a concomitant increase in their biocompatibility compared to the metallic nanoparticles that lacked such protein coatings.

In their study, Tan et al. synthesized titanium dioxide (TiO_2_) nanoparticles and subsequently conjugated the surfaces of these nanoparticles with porous nylon substrates.^[^ ^124^ ^]^ The purpose of this conjugation was to enable selective enrichment of phosphopeptides, which were then subjected to mass spectrometry (MS) analysis. The utilization of this methodology facilitates the comprehensive examination of protein phosphorylation sites and their corresponding patterns, thereby playing a pivotal role in the elucidation of pathological conditions characterized by excessive phosphorylation events.^[^ ^124^, ^125^ ^]^ The present investigation involved conducting in vivo experiments using porous nylon TiO_2_ nanoparticles to elucidate the crucial phosphorylation sites of tau protein in the context of AD progression. The aforementioned findings have the potential to lay the groundwork for the advancement of more targeted and efficient therapeutic interventions aimed at suppressing tau hyperphosphorylation.^[^ ^124^, ^126^, ^127^ ^]^

The investigation of various metallic nanoparticles, such as silver, zinc, and platinum, for their potential application in the treatment of AD has been limited. This was primarily attributed to their inadequate biocompatibility and cytotoxic effects. Iron and titanium nanoparticles have emerged as promising candidates for AD therapy. These nanoparticles have the potential to selectively target tau protein and its phosphorylation processes, thereby offering prospects for therapeutic interventions in AD [See Table 5]. Additional investigations are warranted to substantiate the efficacy and safety of the aforementioned interventions for the treatment of AD in both preclinical and clinical contexts.

**TABLE 5:** Summary of other metallic tau‐targeting nanoparticles for applications in AD treatment.

Nanoparticle type	Objective	Size	Dose administered	Experimental system	Notable results	Reference
Protein‐capped iron oxide and cadmium sulfide nanoparticles	Synthesize a metallic‐based nanoparticle that is effective in preventing tau aggregation and polymerization to function as a potential AD treatment	Protein‐capped iron oxide nanoparticle: 40–50 nm Cadmium sulfide nanoparticle: 5–20 nm	5–100 µg mL^−1^	Neuro2a neuroblastoma cell line	Both synthesized nanoparticles had optimal biocompatibility with the Neuro2a neuroblastoma cell line. Additionally, both nanoparticles served a synergistic effect to prevent tau accumulation and inhibit tau aggregation	[123]
Titanium dioxide nanoparticles conjugated with porous nylon substrates	Design a metallic‐based nanoparticle that functions to provide more insight about the seven in vivo phosphorylation sites that are often hyperphosphorylated in AD	Not specified	Not specified	Not specified	The groundwork for the advancement of more targeted and efficient therapeutic interventions aimed at suppressing tau hyperphosphorylation is established	[124]

## 16 MAGNETIC NANOPARTICLES (MNPS)

The utilization of magnetic nanoparticles (MNPs) has garnered considerable attention in the field of nanomedicine, particularly in the discernment and management of AD.^[^ ^128^ ^]^ The use of MNPs in various applications has garnered significant attention owing to their inherent magnetic properties.^[^ ^113^ ^]^ These properties confer several advantages, including the ability to facilitate controlled drug release, enable efficient drug loading, allow for surface modifications, and exhibit desirable biocompatibility. Recent research has shown that MNPs, specifically, ultra‐small superparamagnetic iron oxide NPs, have significant success in achieving higher circulation times by traveling through capillary walls. Furthermore, MNPs have optimal drug delivery applications due to their ability to work in conjunction with external magnetic fields to act in local regions. Overall, MNP drug delivery systems depend on the forces exerted on the NPs by blood compartment molecules and magnetic forces exerted by the external magnetic field.

MNPs have been employed as sensors for the detection of tau protein in AD diagnosis.^[^ ^113^, ^128^ ^]^ In their seminal work, Demeritte et al. successfully engineered magnetic plasmonic nanoplatforms that exhibited exceptional chemical stabilities.^[^ ^129^ ^]^ These nanoplatforms are specifically designed to facilitate the detection and quantification of amyloid‐beta and tau proteins, even at exceedingly low concentrations in blood samples. Through conjugation techniques, magnetic Fe_3_O_4_ nanoparticles were combined with gold plasmonic shell nanoparticles. Subsequently, these hybrid nanoparticles were bio‐conjugated with 2D graphene oxide coated with antibodies targeting amyloid and tau proteins [see Table 6]. As a result of this experimental approach, researchers have been able to attain notable levels of detection efficiency for both proteins. The nanoplatform exhibited heightened sensitivity compared to conventional enzyme‐linked immunosorbent assay (ELISA) kits, thereby presenting itself as a promising candidate for the detection of early stage AD.

**TABLE 6:** Summary of tau‐targeting magnetic nanoparticles for enhancing AD diagnosis and treatment.

Nanoparticle type	Objective	Size	Dose administered	Experimental system	Notable results	Reference
Graphene‐oxide derived magnetic plasmonic nanoplatforms	Exploit the high sensitivity of high plasmon‐coupling nanoplatforms to develop a more effective diagnostic that can distinguish between AD biomarkers (i.e., tau fibrils and amyloid‐β proteins) and whole blood serum	Not specified	Not specified	Not specified	The graphene‐oxide derived magnetic plasmonic nanoplatforms can identify tau fibril and amyloid‐β concentrations, as low was 500 fg mL^−1^. Additionally, the synthesized nanoplatforms had significantly higher detection limits when compared to conventional enzyme‐linked immune sorbent assays (ELISA)	[129]
Fe‐MIL‐88B‐NH_2_encapsulated with methylene blue (inhibitor of tau aggregation) and functionalized with defluorinated MK6240 and DMK6240	Formulate an advanced drug delivery system that increases the bioavailability of methylene blue, improves hyperphosphorylated tau targeting, enhances magnetic resonance imaging, mitigates neuronal death, and ameliorates AD‐related symptoms	190–240 nm	6.25, 12.5, 25, 50, 100 µg mL^−1^	SH‐SY5Y cells	Encapsulating methylene blue within the magnetic‐derived nanoparticle facilitated its permeations across the BBB and enhanced its bioaccumulation in target tissue. Furthermore, the synthesized NPs were able to effectively target hyperphosphorylated tau, facilitate neuronal stability, decrease inflammation and damage to hippocampal regions. In vivo studies with AD rats that the conjugated NPs improved memory and cognition.	[130]
Magnetic iron‐oxide nanoparticles coated with PEG‐b‐AGE and conjugated with anti‐amyloid‐β and anti‐tau antibodies	Synthesize a nanoparticulate system that offers more sensitive detection of tau and amyloid‐β proteins by limiting nonspecific interactions with increased efficacy than conventional systems	100–130 nm	0.1, 0.5, 2.0, and 10 µg mL^−1^	CSF samples collected by lumbar punctures and whole blood serum samples collected through veins	The synthesized nanoparticles exhibited a 90% improved specificity and a 95% improved sensitivity to amyloid‐β and tau biomarkers than conventional magnetic micron beads, Dynabeads	[131]

The study conducted by Zhao et al. aimed to develop theragnostic magnetic nanoparticles that can target tau proteins, thereby facilitating the diagnosis and treatment of AD.^[^ ^130^ ^]^ The nanoparticles used in this study were constructed using Fe‐MIL‐88B‐NH2 as a base material. These nanoparticles were designed to incorporate Methylene Blue, a well‐known inhibitor of tau aggregation, and to serve as MRI contrast agents. Specific targeting of hyperphosphorylated tau was significantly enhanced through the surface functionalization of targeting agents, namely, MK6240 and DMK6240. Encapsulating methylene blue within the magnetic‐derived nanoparticle facilitated its permeations across the BBB and enhanced its bioaccumulation in target tissue. Furthermore, the synthesized NPs were able to effectively target hyperphosphorylated tau, facilitate neuronal stability, decrease inflammation and damage to hippocampal regions. In vivo studies with AD rats that the conjugated NPs improved memory and cognition. The nanoparticles under investigation have shown promising potential for both diagnostic and therapeutic applications in AD, as evidenced by their successful performance in in vitro and in vivo studies. Specifically, these studies have demonstrated the effective magnetic resonance imaging (MRI) capabilities of nanoparticles, as well as their ability to prevent tau aggregation and neuronal death. These findings highlight the potential of NPs as valuable tools for the detection and treatment of AD.

Li et al. developed a novel approach for early diagnosis by formulating polymer polyethylene glycol‐block‐allyl glycidyl ether (PEG‐b‐AGE) coated magnetic iron‐oxide nanoparticles.^[^ ^131^ ^]^ The nanoparticles exhibited heightened levels of specificity and sensitivity towards amyloid‐beta and tau proteins in both cerebrospinal fluid (CSF) and human blood samples, surpassing the capabilities of existing diagnostic techniques. By excluding interactions with non‐amyloid beta AD protein biomarkers, it is plausible that these nanoparticles can enhance the precision of early AD detection.

In the realm of AD research, MNPs have emerged as a promising avenue with considerable potential in the field of AD research. This class of nanoparticles can revolutionize the field by facilitating advancements in both diagnostic and therapeutic applications. Through continued advancements and rigorous examinations within clinical settings, it is plausible that these instruments may emerge as invaluable assets in the ongoing battle against AD.

## 17 MESOPOROUS SILICA NANOPARTICLES

Mesoporous silica nanoparticles (MSNs) possess many advantages as drug delivery systems. These NPs often have large surface areas, tunable sizes and shapes, multifunctional abilities, and good hemocompatibility in various physiological environments.^[^ ^132^ ^]^ Furthermore, their optimal electrical, magnetic, and optical properties as well as their tunability for size, structure, and geometry render them significant for biomedical therapeutic applications. Additionally, their high drug loading capabilities and feasible surface modifications make them a promising nanotherapeutic option for AD.

In the study by Chen et al., MSNs were surface‐conjugated with ultrasmall ceria nanocrystals (CeNCs) and iron oxide nanocrystals (IONCs) to target hyperphosphorylated tau and inhibit critical pathways of tau‐driven AD pathogenesis.^[^ ^133^ ^]^ The MSNs were loaded with methylene blue (MB), which is an inhibitor of tau aggregation. The nanoparticles were further functionalized with the tau tracer, T807, to optimize their selectivity and binding capabilities to tau aggregates. In vivo studies indicated that CeNC/IONC/MSN‐T807 NPs have enhanced retention in the hippocampus of tauopathy rat models. Further studies demonstrated that the formulated NPs had the ability to significantly reduce mitochondrial reactive oxygen species in vitro. In addition, the synergistic effects of CeNC/IONC/MSN‐T807‐MB NPs resulted in effective inhibition of tau phosphorylation at the protein sites, S396, S19, S404, and T205 and the near‐elimination of tau‐tau binding. The CeNC/IONC/MSN‐T807‐MB NPs were also capable of inhibiting tau aggregation to 13.3%, while MB was capable of inhibiting tau aggregation to only 25%. The group treated with CeNC/IONC/MSN‐T807‐MB NPs exhibited more than 71.8% in cell viability. In contrast, the control group's cell viability was only 23.8% and the MB group's cell viability was 35.2%. The NPs formulated by Chen et al., reduced the neuronal apoptosis rate to 10.34%, while sole MB treatment decreased the neuronal apoptosis rate by merely 23.54%. In vivo studies confirmed that the CeNC/IONC/MSN‐T807‐MB NPs could preserve memory functions by reducing microglial and astrocyte activation. With further studies to increase BBB penetration capabilities, CeNC/IONC/MSN‐T807‐MB NPs could be clinically utilized to treat AD patients.

Sun et al. composed magnetic mesoporous silica nanoparticles (M‐MSN) that are functionalized with ceria nanoparticles (CNPs) and anti‐tau antibodies (AT8) as a potential therapeutic for AD.^[^ ^134^ ^]^ In vitro studies showed the potential for CNPs to promote autophagy and prevent pathogenic tau accumulation. The functionalization of the NPs with AT8 facilitated the binding and accumulation of the NPs to pathological tau phosphorylation sites, such as Ser202/Thr205.^[^ ^134^, ^135^ ^]^ In vivo studies by Sun et al. indicated that the formulated NPs improved neuronal viability and cognitive functions and decreased pathogenic tau burden. These benefits are likely because the formulated NPs reduce microglial activation. Other in vitro and in vivo studies confirmed that the AT8‐CNP‐M‐MSN NPs degraded pathogenic tau by inhibiting the AKT/mTOR signaling pathway. These NPs have also demonstrated high binding affinity for hyperphosphorylated tau, while minimizing interference with other biological proteins [see Table 7].

**TABLE 7:** Summary of tau‐targeting mesoporous silica nanoparticles for applications in AD treatment.

Nanoparticle type	Objective	Size	Dose administered	Experimental system	Notable results	Reference
Methylene‐blue loaded mesoporous silica nanoparticles surface‐conjugated with ultrasmall ceria nanocrystals and iron oxide nanocrystals	Develop an effective nanomaterial‐based interventional system that can suppress tau hyperphosphorylation, limit mitochondrial oxidative stress, inhibit critical AD pathogenic pathways, and mitigate neuronal apoptosis	132 nm	0.0625, 0.125, 0.25, 0.5, 1.0 mm	In vitro: SH‐SY5Y cells In vivo: Tauopathy AD rat models	The synthesized NPs were found to inhibit tau hyperphosphorylation and aggregation, prevent apoptosis of neurons, limit neuroinflammation, and function as a reactive oxygen species scavenger to decrease mitochondrial associated oxidative stress. Furthermore, in vivo studies indicated that the NPs alleviated memory and learning deficits caused by AD	[133]
Mesoporous silica nanoparticles functionalized with ceria‐nanoparticles and anti‐tau antibodies	Synthesize mesoporous silica nanoparticles to promote the clearance of tau aggregates, inhibit tau hyperphosphorylation, facilitate autophagy in neuronal cells, improve neuronal viability, and restore optimal cognitive function AD rat models	79 nm	2.5, 5, 10 µg mL^−1^	In vitro: SH‐SY5Y cells In vivo: AD rat model	The synthesized NPs were found to be successful in achieving biocompatibility, promoting autophagy, clearing tau aggregates, enhancing neuronal viability, and improving cognitive function in AD rats	[134]

## 18 MESOPOROUS CARBON NANOPARTICLES

Mesoporous carbon nanoparticles have gained widespread attention for biomedical therapeutic applications due to their unique electrochemical and optical properties. These properties further facilitate their simple controllability over size, high surface area, and optimal sensitivity and selectivity. The study of mesoporous carbon nanoparticles (MCN) as an option for the administration of sustained drug delivery and targeted therapy presents a compelling prospect in the realm of AD treatment.^[^ ^136^ ^]^ In their study, Xu et al. provided evidence supporting the therapeutic efficacy of oxidized MCN loaded with Protoporphyrin IX (PX)—a specific drug—and functionalized with RVG peptides in the context of AD.^[^ ^137^ ^]^. PX emerges as a compelling contender for the treatment of AD due to its capacity to mitigate tau phosphorylation while exhibiting a notable absence of deleterious toxicological ramifications. Nevertheless, the therapeutic efficacy of the substance is constrained due to its incapacity to traverse the blood‐brain barrier (BBB).

The modifications made to the RVG (rabies virus glycoprotein) on the MCN (magnetic carbon nanotube) surfaces have been observed to enhance the ability of these nanotubes to penetrate the blood‐brain barrier (BBB). This conclusion is supported by both in vitro (performed in a controlled laboratory setting) and in vivo (conducted in living organisms) studies. Furthermore, the utilization of ultrasound radiation was employed to accomplish precise and targeted drug delivery within the intricate confines of the human brain. The in vitro investigations have substantiated the presence of crucial attributes in the synthesized PX‐MCN‐RVG nanoparticles, including their capacity for accumulation, biocompatibility, minimal immunogenicity, and absence of cytotoxic effects. These findings establish the potential efficacy of these nanoparticles for therapeutic applications.

The combination of PX‐MCN‐RVG NPs with ultrasound therapy has demonstrated encouraging outcomes in the context of inhibiting amyloid‐beta monomer aggregation by approximately 71% and impeding amyloid‐beta mediated apoptosis in an in vitro setting. Furthermore, the NPs that were formulated demonstrated effective inhibition of GSK3β‐mediated tau phosphorylation, which is a crucial mechanism involved in the pathogenesis of AD. The efficacy of PX‐MCN‐RVG NPs in the treatment of AD has been substantiated through in vivo studies. These investigations have demonstrated the NPs' capacity to impede the progression of the disease and enhance cognitive functions. This study elucidates the inherent capabilities of mesoporous carbon nanoparticles as a versatile framework for the integration of synergy therapies and the precise administration of therapeutic agents, thereby presenting a groundbreaking avenue for mitigating the symptoms associated with AD and laying the groundwork for future progressions in the realm of AD treatment methodologies.^[^ ^137^ ^]^

## 19 FINAL REMARKS: A COMPARATIVE ANALYSIS OF TAU‐TARGETING NANOPARTICLES IN THE CONTEXT OF ALZHEIMER'S DISEASE (AD)

Nanoparticles with tau‐targeting capabilities could encapsulate therapeutic medicines, providing protection from enzymatic degradation. This protective mechanism results in the prolongation of the agents' half‐life within the central nervous system (CNS). The therapeutic potential of this subject is multifaceted. The nanoparticles have the potential to be engineered in a manner that allows for the simultaneous delivery of various therapeutic drugs, which could result in the emergence of synergistic therapeutic effects.

When comparing tau‐targeting nanoparticles to previously utilized therapy tactics and current therapeutic approaches for Alzheimer's disease (AD), it becomes evident that the former presents a number of notable benefits, as outlined below:

- In relation to cholinesterase inhibitors (ChEIs): Tau‐targeting nanoparticles are designed to specifically target the underlying pathogenic mechanisms of AD, while cholinesterase inhibitors (ChEIs) largely offer symptomatic relief without exerting any influence on the course of the illness.
- In relation to NMDA receptor antagonists (NMDA‐Rs): Tau‐targeting nanoparticles are designed to specifically address pathogenic pathways associated with a certain condition, while NMDA‐Rs (N‐methyl‐d‐aspartate receptors) have a broader range of actions that may not exhibit selectivity towards AD.
- In relation to therapies targeting amyloid: The nanoparticles designed to target tau proteins interact with a corresponding pathogenic pathway, which may enhance the effectiveness of medicines that specifically target amyloid proteins when used together.

## 20 CONCLUSION

In summary, it can be inferred that AD continues to pose a significant obstacle in the field of neuroscience, with the absence of viable therapeutic interventions capable of impeding or reversing the trajectory of the disease. Existing therapeutic approaches predominantly emphasize the management of symptoms, thereby necessitating a shift towards investigating experimental pharmaceuticals and targeted interventions as potential curative measures.

However, it is crucial to recognize that the advancement of nanoparticles that target tau is currently in its early phases, requiring additional research to thoroughly evaluate their safety, effectiveness, and long‐term consequences. Moreover, the task of optimizing nanoparticle design, distribution techniques, and targeting precision continues to pose significant challenges. Despite the aforementioned obstacles, it is indisputable that nanoparticles designed to target tau proteins exhibit significant potential in the field of Alzheimer's disease therapies. These nanoparticles have the capacity to provide more effective and accurately directed treatment alternatives. Continual research efforts are focused on overcoming these obstacles and transforming the envisioned capabilities of tau‐targeting nanoparticles into concrete clinical implementations.

## CONFLICT OF INTEREST STATEMENT

The authors declare no conflicts of interest.

## Supporting information

Supporting Information
